# Genome-Wide Identification and Functional Characterization of the Acyl-CoA Dehydrogenase (ACAD) Family in *Fusarium sacchari*

**DOI:** 10.3390/ijms26030973

**Published:** 2025-01-24

**Authors:** Quan Zeng, Quan Yu, Yingxi Mo, Haoming Liang, Baoshan Chen, Jiaorong Meng

**Affiliations:** 1 State Key Laboratory for Conservation and Utilization of Subtropical Agro-Bioresources, Ministry and Province Co-Sponsored Collaborative Innovation Center for Sugarcane and Sugar Industry, Guangxi University, Nanning 530004, China; 1908401002@st.gxu.edu.cn; 2 College of Life Science and Technology, Guangxi University, Nanning 530004, China; 3 Guangxi Key Laboratory of Sugarcane Biology, Academy of Sugarcane and Sugar Industry, College of Agriculture, Guangxi University, Nanning 530004, China; 2017304028@st.gxu.edu.cn (Q.Y.); 1917304014@st.gxu.edu.cn (Y.M.); 2017392018@st.gxu.edu.cn (H.L.)

**Keywords:** *Fusarium sacchari*, Pokkah boeng disease, acyl-CoA dehydrogenase, fatty acid β-oxidation, functional analysis

## Abstract

*Fusarium sacchari* is one of the primary causal agents of Pokkah boeng disease (PBD), an important disease of sugarcane worldwide. The acyl-CoA dehydrogenases (ACADs) constitute a family of flavoenzymes involved in the β-oxidation of fatty acids and amino acid catabolism in mitochondria. However, the role of ACADs in the pathogenesis of *F. sacchari* is unclear. Here, 14 ACAD-encoding genes (*FsACAD-1*–*FsACAD-14*) were identified by screening the entire genome sequence of *F. sacchari*. The *FsACAD* genes are distributed across seven chromosomes and were classified into seven clades based on phylogenetic analysis of the protein sequences. In vivo mRNA quantification revealed that the *FsACAD* genes are differentially expressed during sugarcane infection, and their expression patterns differ significantly in response to the in vitro induction of fatty acids of different classes. Fatty acid utilization assays of the *FsACAD*-deletion mutants revealed that the FsACADs varied in their preference and ability to break down different fatty acids and amino acids. There was variation in the adverse impact of *FsACAD*-deletion mutants on fungal traits, including growth, conidiation, stress tolerance, and virulence. These findings provide insights into the roles of *FsACADs* in *F. sacchari*, and the identification of *FsACADs* offers potential new targets for the improved control of PBD.

## 1. Introduction

Fatty acids play important roles in cell functions, such as energy metabolism, membrane formation, and cell signaling [1,2]. In animals, the oxidation of fatty acids occurs in multiple locations within the cell, e.g., β-oxidation occurs only in the mitochondria, while both α- and β-oxidation occur in the peroxisome, and ω-oxidation occurs in the endoplasmic reticulum [3]. The β-oxidation of fatty acids is a central metabolic process for providing electrons to the respiratory chain to generate energy for aerobic organisms. β-Oxidation is the principal pathway in fatty acid degradation and an important source of energy and carbon skeletons in organisms [4,5]. Acetyl-CoA is a final product of fatty acid β-oxidation [6]. In mammalian cells, mitochondrial β-oxidation is mainly used to generate energy, while peroxisomal β-oxidation is used in the chain shortening of numerous lipophilic compounds for a variety of metabolic processes [7,8]. Only the peroxisomal β-oxidation pathway is present in yeast, while two β-oxidation pathways (mitochondrial and peroxisomal) have been reported in other non-yeast fungi [9,10]. Filamentous fungi can use fatty acids as a carbon source for energy production through the β-oxidation pathway. Although various organelles are involved in the degradation of fatty acids, the degradation of long-chain fatty acids generally occurs in mitochondria [11].

Sugarcane is an important industrial crop, accounting for nearly 70% of the sugar produced worldwide [12]. In China, cane sugar production reached 9.01 million tons in 2019–2020, accounting for 86.7% of the total sugar produced [13]. Pokkah boeng disease (PBD) is one of the most serious fungal diseases affecting most sugarcane-producing areas worldwide [14], reducing cane yield by 40–60% in susceptible sugarcane cultivars. PBD can be caused by several *Fusarium* species, including *F. sacchari*, *F. proliferatum*, *F. andiyazi*, *F. verticillioides*, *F. fujikuroi*, and *F. subglutinans* [15,16,17,18]. Lin et al. reported *F. verticillioides* and *F. proliferatum* as the causative agents of PBD in the sugarcane-producing regions in China, and Meng et al. later demonstrated that *F. sacchari* is the dominant species responsible for the disease in Guangxi province [18,19].

Fatty acids, both free and as part of complex lipids, play key roles in metabolism. They serve as a major metabolic fuel (storage and transport of energy), are essential components of membranes, and act as regulators of gene expression [20,21]. Acyl-CoA dehydrogenases (ACADs) are flavoenzymes involved in the catabolism of fatty acids and amino acids, antibiotic biosynthesis, stress response, and cholesterol metabolism [22]. These enzymes transfer electrons from fatty acids to electron transfer flavoprotein (ETF) for oxidation in mitochondria. This is distinct from acyl-CoA oxidase (ACO) in peroxisomes, which directly oxidizes fatty acids using molecular oxygen [23,24]. ACADs are involved in the first step of the mitochondrial β-oxidation pathway [6]. In humans, there are eleven types of ACAD proteins classified into three categories: five involved in the β-oxidation of fatty acids, four involved in the catabolism of amino acids, and two with unknown functions [23,24]. With the exception of very-long-chain acyl-CoA dehydrogenase, all proteins of the ACAD superfamily are found in the form of soluble homo tetramers with a subunit mass of approximately 43 kDa, and each subunit contains one flavin adenine dinucleotide (FAD) [23]. Investigations on the human pathogen *Mycobacterium tuberculosis* have revealed that inhibition of the fatty acid β-oxidation process mediated by ACADs results in loss of the capacity to metabolize fatty acids and compromised viability in murine hosts for the pathogen [25]. In the phytopathogenic fungus *Magnaporthe oryzae*, ACADs have been shown to have varied effects on growth, conidial germination, and pathogenicity [26]. However, little is known about the function of ACADs in *F. sacchari*.

In this study, we identified the ACAD-encoding genes in *F. sacchari* and investigated their functions using a gene deletion strategy. We found that the *FsACADs* are differentially involved in the regulation of fungal growth and development, stress response, nutrient absorption, and virulence. The findings enrich the understanding of the molecular mechanism of fungal ACADs and provide potential targets for PBD control.

## 2. Results

### 2.1. Identification of Acyl-CoA Dehydrogenase Genes in F. sacchari

By searching the non-redundant (NR) protein sequence database and Pfam database of *F. sacchari* FF001 (Accession No. PRJCA031149), we identified 14 ACAD encoding genes, designated *FsACAD-1* to *FsACAD-14* (Table 1).

### 2.2. Gene Organization in the FsACAD Family

**Figure 1:**
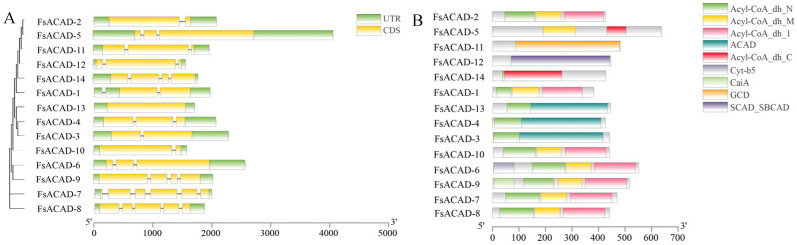
Visualization of gene structures. (**A**) Gene structures of the *Fusarium sacchari acyl-CoA dehydrogenases* (*FsACADs*). Septal lines indicate introns and other structures are exons except for the UTR on both ends. (**B**) Protein domain prediction of FsACADs using NCBI CDD (Conserved Domain Database).

The intron distribution of the *FsACAD* genes was verified by cDNA cloning and sequencing and visualized using TBtools [27]. Except for *FsACAD-13*, which is intron-free, all *FsACADs* contain one to five introns (Figure 1A). Protein domain analysis showed that two of the FsACADs each harbor one type of domain (SCAD-SBCAD, 1; GCD, 1), four FsACADs harbor two types of domains (Acyl-CoA_dh_N + Acyl-CoA_dh_C, 1; Acyl-CoA_dh_N + ACAD, 3), there are five FsACAD proteins with three types of domains (Acyl-CoA_dh_N + Acyl-CoA_dh_M + Acyl-CoA_dh_l) and two FsACADs with four domains (one Cyt-b5 or one CaiA plus three others) (Figure 1B). The structure prediction revealed that all ACAD proteins have similar tertiary structures (Figure S1).

### 2.3. Phylogenetic Relationship Among ACAD Proteins

**Figure 2:**
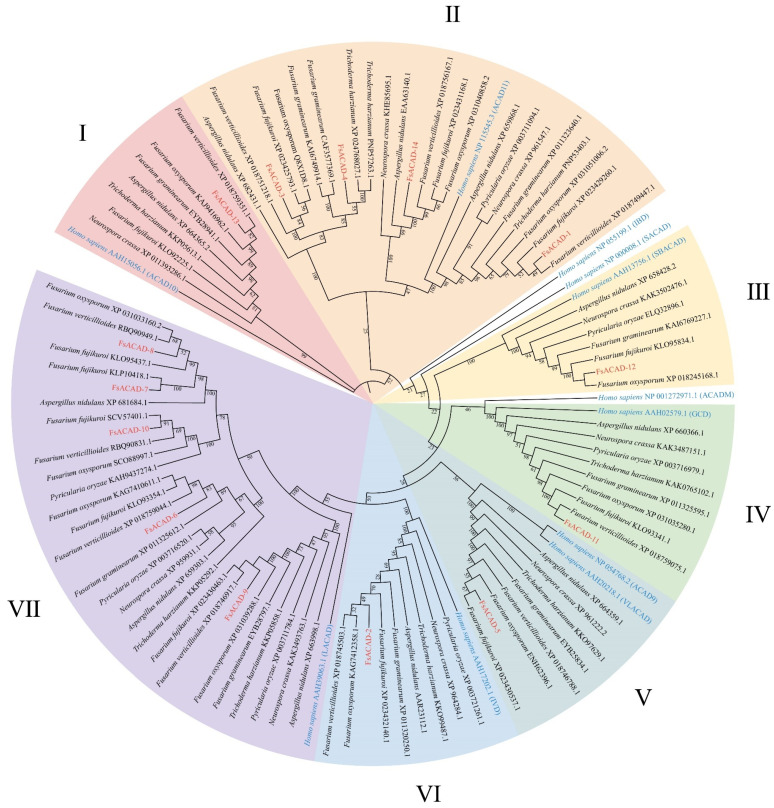
Phylogenetic relationship of acyl-CoA dehydrogenases (ACADs). The phylogenetic tree was constructed using the maximum likelihood method with 5000 bootstraps and adjusted using the online tool iTOL. FsACADs are marked in red, and ACADs from *Homo sapiens* are marked in blue. Reference to *Homo sapiens*, the phylogenetic tree was delineated seven groups (I to VII), encompassing ACAD10, ACAD11, SBACAD (Short/branched-chain acyl-CoA dehydrogenase), GCD (Glutaryl-CoA dehydrogenase), ACAD9/VLACAD, IVD (Isovaleryl-CoA dehydrogenase), and LACAD (long-chain-specific acyl-CoA dehydrogenase).

A phylogenetic tree was constructed based on FsACAD proteins and 91 ACAD proteins from various sources, including humans and eight other species from five genera of filamentous fungi, such as *Fusarium fujikuroi*, *Fusarium verticillioides*, *Fusarium oxysporum*, *Fusarium graminearum*, *Pyricularia oryzae*, *Neurospora crassa*, *Aspergillus nidulans*, and *Trichoderma harzianum*. Seven groups (I to VII) were generated in the tree (Figure 2). Since none of the fungal species seems to harbor more than ten ACADs, we used *Homo sapiens* as a reference for comparing their ACADs with those from *F. sacchari*. There are three independent branches that are only present in *Homo sapiens* (isobutyryl-CoA dehydrogenase, IBD; short-chain-specific acyl-CoA dehydrogenase, SACAD; and medium-chain-specific acyl-CoA dehydrogenase, ACADM) and therefore not in filamentous fungi. We also noticed that most of the ACADs from *F. sacchari* were distributed in group VII (five members) and three in group II. In contrast, there is only one member in each of the other groups (I, III, IV, V, IV) (Figure 2). Both ACAD9 and VLACAD (very long-chain-specific acyl-CoA dehydrogenase) in *Homo sapiens* are in group V. Functional annotation reveals that group VII is composed of long-chain acyl-CoA dehydrogenases. Multiple ACAD members present in this group suggest that the utilization of long-chain fatty acids is vital in *F. sacchari*.

### 2.4. FsACAD Gene Expression Patterns During Sugarcane Infection

**Figure 3:**
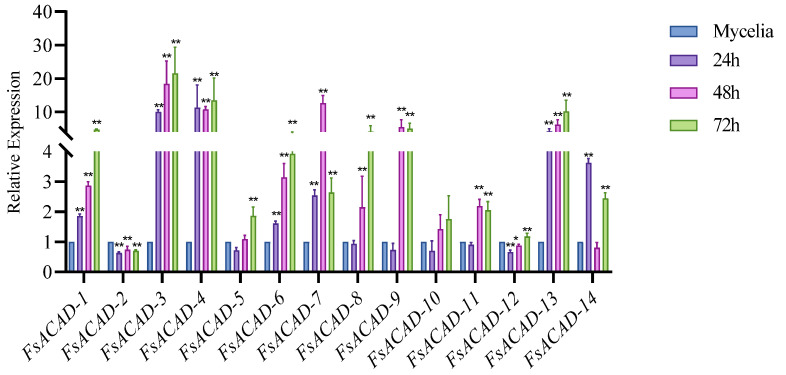
Expression of *Fusarium sacchari* acyl-CoA dehydrogenase genes during infection. *Fusarium sacchari* FF001 was inoculated onto sugarcane leaves for 24, 48, and 72 h, and the relative expression levels of each *ACAD* gene were measured. Mycelium of in vitro culture was used as the control. Each treatment consists of three biological replicates, of which there are three technical replicates. Differential analysis was performed using SPSS, * indicates *p* < 0.05; ** indicates *p* < 0.01.

To investigate the expression patterns of *FsACADs* during sugarcane infection, detached sugarcane leaves from plants at the sixth–seventh leaf stage were inoculated with fungal mycelium. qRT-PCR was conducted at infection stages of 24, 48, and 72 h post-inoculation (hpi). The expression levels of *FsACAD-1*, *FsACAD-3*, *FsACAD-4*, *FsACAD-6*, *FsACAD-7*, and *FsACAD-13* were found to be consistently upregulated during the entire infection process when compared to the mycelium prior to inoculation. While *FsACAD-8*, *FsACAD-9*, and *FsACAD-11* expression sharply increased at 48 hpi and maintained at high levels until 72 hpi, *FsACAD-14* was upregulated at 24 hpi and 72 hpi but downregulated at 48hpi. In contrast, the expression level of *FsACAD-2* continuously decreased from 24 hpi to 72 hpi (Figure 3).

### 2.5. Expression of FsACADs Is Differentially Induced by Different Fatty Acids

**Figure 4:**
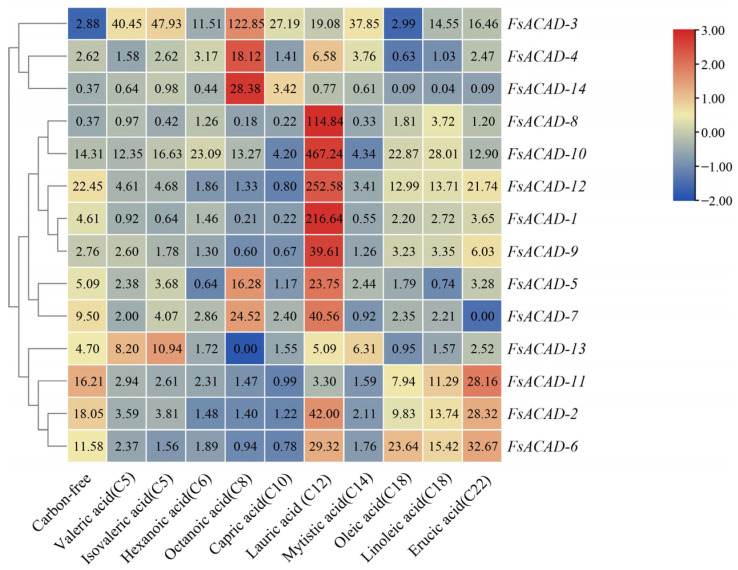
Heatmap of *Fusarium sacchari acyl-CoA dehydrogenase* genes expression induced by fatty acids with different carbon chain lengths. The expression of *FsACADs* on the sucrose-based medium was used as the control, and *FsACAD* expression was induced using different fatty acids as the sole carbon source. The heatmap was generated using the logarithm of the relative expression values (log10 value). The y-axis represents *FsACADs,* and the x-axis represents different carbon sources. The figure in the blocks is the original value of relative expression levels (2^−ΔΔCT^). The color bar on the right shows the expression level as a log10 exponent. In sucrose-based culture settings, the reference value of 0 for the *FsACAD* expression level was established following log10 transformation from the original value of 1. Blue indicates downregulation, and red indicates upregulation.

qRT-PCR was employed to investigate the expression patterns of the *FsACAD* genes in liquid media with different fatty acids as the sole carbon source. The expression level of the *FsACAD* genes in a medium with sucrose as the sole carbon source was set to a value of 1 and served as the control. As shown in Figure 4, all *FsACAD* genes except for *FsACAD-8* and *FsACAD-14* were upregulated 2.6- to 22.5-fold (ratio of 2^−ΔΔCT^ value) under sucrose deprivation when compared to expression levels in medium with sucrose as the carbon source. This upregulation might possibly be due to the mobilization of cellular fatty acids for energy production within the cells.

The expression of all 14 *FsACADs* was induced by at least one type of fatty acid. While expression in 13 of the 14 *FsACADs* (*FsACAD-1* to *FsACAD-13*) was induced by lauric acid, *FsACAD-3* could be induced by all of the fatty acids tested. However, a clear specificity between *FsACADs* and fatty acids was observed: *FsACAD-14* was induced by octanoic acid and capric acid but suppressed by all other fatty acids; *FsACAD-8* was induced by lauric acid and linoleic acid but suppressed by myristic acid, valeric acid, isovaleric acid, octanoic acid, and capric acid. Furthermore, *FsACAD-2*, *FsACAD-6*, and *FsACAD-11* were preferentially induced by long-chain fatty acids (Figure 4).

### 2.6. FsACADs Differentially Impact Hyphal Growth, Conidiation, and Stress Response

**Figure 5:**
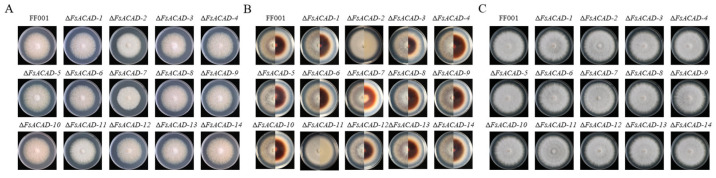
Growth of mutants of *Fusarium sacchari* acyl-CoA dehydrogenase genes in different media. (**A**) front view of colonies on PDA; (**B**) colonies on MM. Front view (left) and back view (right) are presented for each colony; (**C**) front view of colonies on CM. Images were taken five days after cultivation. The petri dish is 90 mm in diameter.

Impact of *FsACADs* on hyphal growth. To explore the effect of different *FsACADs* on the growth and development of the fungus, deletion mutants of a single *FsACAD* gene were constructed using homologous recombination and evaluated on solid media of potato dextrose agar (PDA), minimal medium (MM), and complete medium (CM). Compared with the wild-type strain FF001, slower growth rates (70.47% to 97.48% of the wild type, *p* < 0.05) were observed in Δ*FsACAD-2*, Δ*FsACAD-3*, Δ*FsACAD-4*, Δ*FsACAD-8*, Δ*FsACAD-9*, Δ*FsACAD-11*, and Δ*FsACAD-12* on all three media, with Δ*FsACAD-2* being the most affected; slower growth rates (92.28% to 97.48% of the wild type) were observed in Δ*FsACAD-1*, Δ*FsACAD-5*, Δ*FsACAD-6*, and Δ*FsACAD-13* on PDA and MM but not on CM. Δ*FsACAD-7* grew slowest on PDA (67.35%) and slower on CM (76.04%) but faster (102.56%) on MM. Δ*FsACAD-10* grew faster on MM and CM; Δ*FsACAD-14* grew faster on PDA and MM but slower on CM (Table 2, Figure 5). These results demonstrate the complex roles of FsACADs in nutrition acquisition and the promotion of fungal growth.

FF001 developed a dark brown pigment on MM three days after cultivation. Enhanced pigment production was observed in Δ*FsACAD-3* and Δ*FsACAD-12*, while reduced pigment production was observed in Δ*FsACAD-7* and Δ*FsACAD-14*, and no pigment was produced in Δ*FsACAD-2* and Δ*FsACAD-11* (Figure 5B). Increased density of aerial hyphae was seen in Δ*FsACAD-2*, Δ*FsACAD-7*, and Δ*FsACAD-11* (Figure 5). Analysis of the mycelial dry weight revealed that under MM and CM culture conditions, the mycelial biomass of the Δ*FsACAD-2* and Δ*FsACAD-11* mutants exceeded that of FF001 (Table S1).

Impact of *FsACADs* on conidiation. We set an arbitrary system to grade the impact of *FsACADs* on conidiation in reference to the sporulation level of the wild-type FF001, i.e., high (≥3-fold), medium (>1.5–<3.0-fold), and marginal (<1.5-fold). Compared to the wild-type strain (3.95 × 10^6^ spores/cm^2^) on PDA, a significantly lower level of sporulation was seen in Δ*FsACAD-2* and Δ*FsACAD-11*, with 6.6-fold and 5.4-fold reduction in sporulation. A medium impact was observed in Δ*FsACAD-7*, and sporulation was marginally impacted in the remaining mutants. On MM, the conidial yield of the wild-type strain was 3.69 × 10^6^ spores/cm^2^. The impact on sporulation was positive medium in five mutants (Δ*FsACAD-1*, Δ*FsACADI-4*, Δ*FsACAD-5*, Δ*FsACAD-7*, and Δ*FsACAD-13*), negative medium for three mutants (Δ*FsACAD-2*, Δ*FsACAD-11*, and Δ*FsACAD-13*), and marginal for the remaining mutants. On CM, the conidial yield of the wild-type strain was 5.81 × 10^6^ spores/cm^2^. Δ*FsACAD-2* and Δ*FsACAD-11* had much lower sporulation levels (4.1-fold and 7.5-fold reduction), and Δ*FsACAD-7*, Δ*FsACAD-10*, and Δ*FsACAD-13* had 1.59- to 1.87-fold reductions in sporulation. The remaining mutants only showed a marginal difference compared to the wild-type strain (Table 3).

As seen in Table 3, Δ*FsACAD-2* produced conidia at a significantly lower level on all three media, accounting for 15.1%, 43.1%, and 24.4% of the spores of the wild-type FF001 on PDA, MM, and CM, respectively. Likewise, Δ*FsACAD-11* yielded 18.6%, 42.3%, and 13.3% of the spores of FF001 on PDA, MM, and CM. Conversely, enhancement in sporulation was observed in Δ*FsACAD-5*, 176.2% on PDA and 197.1% on MM compared with the FF001. Compared with FF001, Δ*FsACAD-7* produced significantly fewer spores on PDA (57.7%) but more spores on MM (273.7%), whereas Δ*FsACAD-9* yielded 127.6% on PDA, 122.3% on MM, but only 53.5% on CM. These results suggest there is a complex interaction between the *FsACADs* and the type of medium.

**Figure 6:**
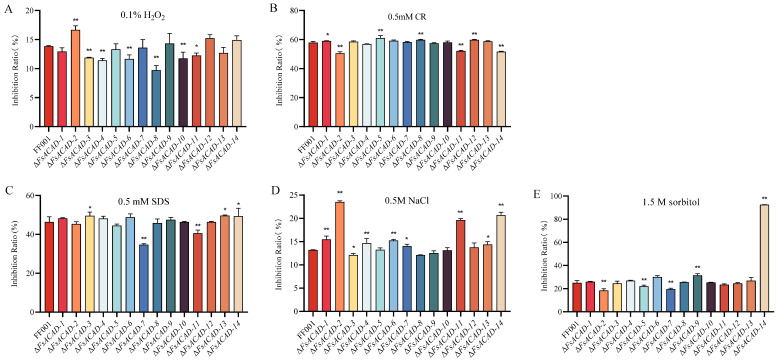
Growth phenotype of *Fusarium sacchari* acyl-CoA dehydrogenase gene mutants under stress conditions. (**A**–**E**) Inhibition rates of FF001 and *FsACAD* mutant strains on CM plates with 0.1% H_2_O_2_, 0.5 mM CR, 0.5 mM SDS, 0.5 M NaCl, and 1.5M sorbitol. Error bars represent standard deviation, and asterisks indicate significant differences in inhibition ratio between FF001 and mutant strains (* *p* < 0.05; ** *p* < 0.01).

Contribution of *FsACADs* to stress tolerance. Mutant strains were exposed to stress environments by cultivation on CM, to which various stressors were added. Under oxidative stress (0.1% H_2_O_2_), the inhibition rate of Δ*FsACAD-2* was 16.7%, higher than the wild-type FF001 (13.9%), suggesting that this ACAD is involved in the eradication of reactive oxidative species (ROS). Of interest, six *FsACADs* (*FsACAD-3*, *FsACAD-4*, *FsACAD-6*, *FsACAD-8*, *FsACAD-10*, and *FsACAD-11*) appear to function in enhancing the fungal sensitivity to ROS stress, as knockout of these genes resulted in lower inhibition rates (Figure 6A). When exposed to the cell wall integrity inhibitor Congo red (CR), opposing effects were observed for the *FsACADs*, i.e., Δ*FsACAD-2*, Δ*FsACAD-11*, and Δ*FsACAD-14* were more tolerant (50.6–52.1% inhibition rate, *p* ≤ 0.05) and Δ*FsACAD-1*, Δ*FsACAD-5*, Δ*FsACAD-8*, and Δ*FsACAD-12* were more sensitive (59.1–61.1% inhibition rate) (Figure 6B). When stressed using the detergent sodium dodecyl sulfate (SDS), Δ*FsACAD-7* and Δ*FsACAD-11* were inhibited to levels (34.7% and 40.6% lower than the wild type (46.4%), while Δ*FsACAD-3*, Δ*FsACAD-13*, and Δ*FsACAD-14* had higher inhibition rates, 49.5–49.7% (Figure 6C). Significant growth inhibition (15.5%, 23.5%, 15.3%, 19.6%, and 20.7%) was observed in Δ*FsACAD-1*, Δ*FsACAD-2*, Δ*FsACAD-6*, Δ*FsACAD-11*, and Δ*FsACAD-14*, respectively, as compared with the inhibition rate of 13% for the wild type, when stressed using 0.5 M NaCl (Figure 6D). The most profound inhibition of 92.5% was seen in Δ*FsACAD-14* when stressed using 1.5 M sorbitol. For this osmotic stress condition, the inhibition rate was 25.1% for the wild type and 18.5% to 31.5% for the remaining *FsACAD* mutants (Figure 6E). These findings suggest that the different FsACADs play varied roles in responding to a range of stresses, either positively or negatively regulating stress response (Figure 6 and Figure S2).

### 2.7. FsACADs Have Varied Impact on Fungal Utilization of Fatty Acids

**Figure 7:**
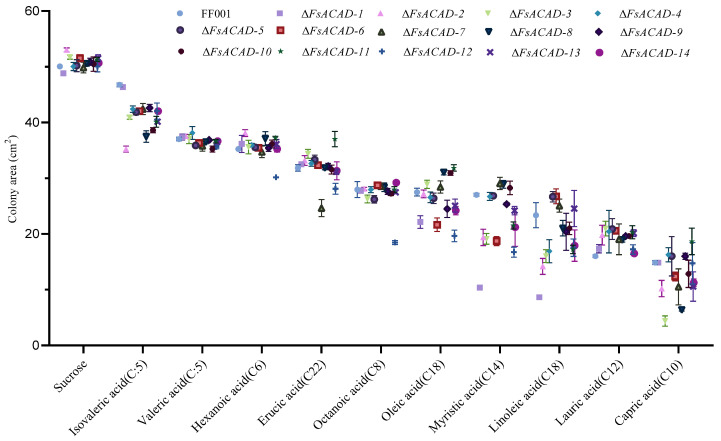
Utilization of fatty acids by *Fusarium sacchari* acyl-CoA dehydrogenase gene mutants. The mutants were cultured in MM supplemented with fatty acids of varying carbon chain lengths, which served as a substitute for sucrose as the carbon source. The *FsACAD* mutants are each represented by dots of different shapes. The number of carbons is indicated in the parentheses. Colony size was measured 5 days after cultivation. Some errors cannot be represented as they are smaller than the symbol used to indicate them. *p* < 0.05.

As judged by colony size, sucrose was the best carbon source for wild-type FF001 (colony size = 50.06 cm^2^), surpassing all other fatty acids tested. When the carbon source was restricted to fatty acids, the suitability of the fatty acids for FF001 followed the order isovaleric acid (93.4%) > valeric acid (74.0%) > hexanoic acid (70.4%) > erucic acid (63.6%) > octanoic acid (55.9) > oleic acid (54.9%) > myristic acid (54%) > linoleic acid (46.7%), lauric acid (32.0%) > capric acid (29.7%) (Figure 7 and Figure S3). The wide range of variation in the efficiency of fatty acid utilization suggests that the fungus possesses different enzymatic activities toward particular fatty acids. To evaluate the impact of individual *FsACADs* on the utilization of different fatty acids, *FsACAD* mutants were cultured on MM plates supplemented with various fatty acids as the sole carbon source.

In the medium with isovaleric acid as the sole carbon source, the wild-type strain FF001 grew almost as well as in the medium with sucrose as the sole carbon source, suggesting the fungus possesses a highly efficient system to metabolize this fatty acid. Compared with the wild type, the colony size of Δ*FsACAD-2* was the smallest (24.6% reduction), followed by Δ*FsACAD-8* (19.8%) and Δ*FsACAD-10* (17.4%), while the remaining *FsACAD* mutants were 8.9% to 14.2% reduced in colony size. These results suggest Δ*FsACAD-2*, Δ*FsACAD-8*, and Δ*FsACAD-10* contribute dominantly and the other *FsACADs* minorly to β-oxidization of isovaleric acid (Figure 7 and Table S2).

The colony size of the wild type was 26% smaller in medium with valeric acid rather than sucrose as the sole carbon source. Of the 14 *FsACAD* mutants, 5 (Δ*FsACAD-5*, Δ*FsACAD-6*, Δ*FsACAD-7*, Δ*FsACAD-10*, and Δ*FsACAD-12*) yielded statistically smaller colonies than the wild type, with reduction rates ranging from 1.9% to 4.8% (Figure 7 and Table S2). Though these *FsACADs* collectively contribute to the oxidization of valeric acid, their enzymatic activity does not seem sufficient to ensure the vigorous growth of the wild-type strain in the medium with valeric acid as the sole carbon source. It may well be that other enzymes are involved in catalyzing the degradation of tryptophan in *F. sacchari*.

The colony size of the wild type was 29.6% smaller in medium with hexanoic acid rather than sucrose as the sole carbon source. Δ*FsACAD-12* was the only mutant with a colony size smaller than that of the wild type (14.35% reduction) (Figure 7 and Table S2).

The wild type exhibited a 36.4% reduction in colony size in the medium with erucic acid compared with sucrose as the sole carbon source. Compared with the wild type on the same medium, Δ*FsACAD-7* had the smallest colonies, with a 22.6% reduction in size, followed by Δ*FsACAD-12*, with an 11.7% reduction, while the remaining mutants showed marginal differences (Figure 7 and Table S2). These results suggest that *FsACAD-7* and *FsACAD-12* are the two major enzymes contributing to the β-oxidization of erucic acid.

The wild type exhibited a 44.1% reduction in colony size in medium with octanoic acid compared with that in medium with sucrose as the sole carbon source. Δ*FsACAD-12* was significantly smaller than the wild type in colony size (34% reduction), followed by Δ*FsACAD-5* (6.3% reduction) and Δ*FsACAD-3* (6.26% reduction) (Figure 7 and Table S2). These results demonstrate that FsACAD-12 is the essential enzyme in *F. sacchari* for metabolizing octanoic acid.

The wild type exhibited a 45.1% reduction in colony size in the medium with oleic acid compared with sucrose as the sole carbon source. Compared with the wild type, Δ*FsACAD-1* (36.4% reduction), Δ*FsACAD-12* (28.9% reduction), and Δ*FsACAD-6* (21.2%) had the smallest colony sizes, followed by Δ*FsACAD-11* (12% reduction) and Δ*FsACAD-9* (11% reduction). Thus, FsACAD-1, FsACAD-12, and FsACAD-6 are the three essential enzymes involved in β-oxidization of oleic acid (Figure 7 and Table S2).

The wild type exhibited a 46% reduction in colony size in medium with myristic acid compared with sucrose utilized as the sole carbon source. Seven *FsACAD* mutants exhibited a more than 20% reduction in colony size as compared with the wild type. Of these mutants, Δ*FsACAD*-*1* was the most affected, with a 63% reduction in growth, followed by Δ*FsACAD-12* (38% reduction) and Δ*FsACAD-6* (30.7% reduction). Of interest, four mutants, Δ*FsACAD-3*, Δ*FsACAD-2*, Δ*FsACAD-14*, and Δ*FsACAD-11*, had reductions of 29.3%, 28.3%, 21.6%, and 20.4%, respectively (Figure 7 and Table S2). Thus, FsACAD-1 is the most important enzyme for β-oxidization of linoleic acid, and FsACAD-12, FsACAD-6, and FsACAD-11 are also essential.

**Figure 8:**
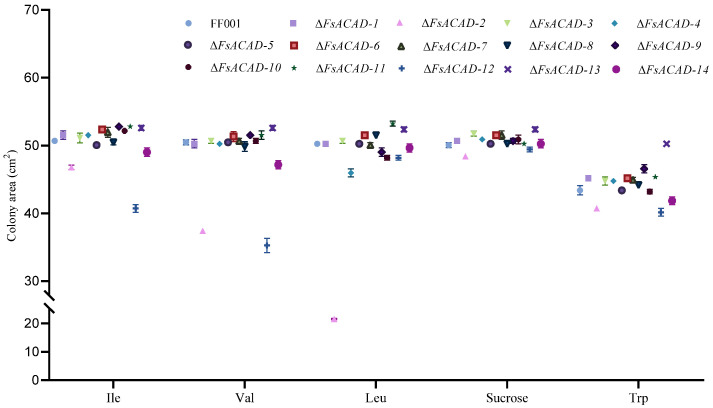
Utilization of amino acids by *Fusarium sacchari* acyl-CoA dehydrogenase gene mutants. The mutants were cultured in MM supplemented with amino acids to replace sucrose as the carbon source. The *FsACAD* mutants are each represented by dots of different shapes. Colony size was measured 5 days after cultivation. Some error bars cannot be represented as they are smaller than the size of the symbol used to indicate them. *p* < 0.05.

The wild type showed a 53.3% reduction in colony size as in medium with linoleic acid compared with sucrose as the sole carbon source. Compared to the wild type, Δ*FsACAD*-*1* was the most affected in growth, with a 63.01% reduction, followed by Δ*FsACAD-2* (39.3% reduction), Δ*FsACAD-3* (31.3% reduction), Δ*FsACAD-4* (27.6% reduction), Δ*FsACAD-11* (26.2% reduction), Δ*FsACAD-12* (25% reduction), and Δ*FsACAD-14* (23.3% reduction) (Figure 8 and Table S2). Thus, FsACAD-1 is the most important enzyme for beta-oxidization of linoleic acid, and FsACAD-2, FsACAD-3, FsACAD-4, FsACAD-11, FsACAD-12, and FsACAD-14 are also essential.

The wild type showed a 68% reduction in colony size in medium with lauric acid compared with sucrose as the sole carbon source. Compared to the wild type, no reduction in colony size was observed in any of the *FsACAD* mutants. Instead, an increase of 22.23% to 30.64% in colony size was observed in nine Δ*FsACADs* (Figure 7 and Table S2). These results indicate that none of the assayed FsACADs are actually involved in lauric acid utilization in *F. sacchari*.

The wild type showed a 70.3% reduction in colony size in medium with capric acid compared with that in medium with sucrose as the sole carbon source. Compared to the wild type, Δ*FsACAD-2* exhibited a 71.7% and Δ*FsACAD-7* a 57.1% reduction in colony size, followed by Δ*FsACAD-1* (31.3%), Δ*FsACAD-6* (29.2%), Δ*FsACAD-12* (28.9%), and Δ*FsACAD-13* (24.3%) (Figure 7 and Table S2). These results indicate that FsACAD-2 and FsACAD-7 are the two most important enzymes for beta-oxidization of capric acid in *F. sacchari*, and FsACAD-1, FsACAD-6, FsACAD-12, and FsACAD-13 are also essential.

### 2.8. FsACADs Have Varied Impact on Utilization of Amino Acids by F. sacchari

Using sucrose as a reference, the wild-type FF001 exhibited high efficiency in the utilization of amino acids as the carbon source. As shown in Figure 8, *F. sacchari* colonies in media with isoleucine (50.7 cm^2^), valine (50.5 cm^2^), and leucine (50.3 cm^2^) were basically the same size as with sucrose (50.1 cm^2^), although slightly smaller in medium with tryptophan (43.4 cm^2^) as the carbon source. To evaluate the contribution of individual FsACADs to the utilization of different amino acids, *FsACAD* mutants were cultured on MM plates supplemented with various amino acids as the sole carbon source.

In a medium with isoleucine as the sole carbon source, reductions in colony size of 19.69% in Δ*FsACAD-12*, 7.75% in Δ*FsACAD-2*, and 4.0% in Δ*FsACAD-14* were observed, while the remaining *FsACAD* mutants had similar colony sizes as the wild-type strain (Figure 8 and Figure S4, Table S3). Thus, FsACAD-12 is the main enzyme for metabolizing isoleucine in *F. sacchari*.

In the medium with valine as the sole carbon source, reductions in colony size of 30.17% in Δ*FsACAD-12*, 25.96% in Δ*FsACAD-2*, and 6.96% in Δ*FsACAD-14* were observed. The remaining *FsACAD* mutants had colony sizes similar to those of the wild-type strain (Figure 8 and Figure S4, Table S3). These results confirm that FsACAD-12 and FsACAD-2 are primarily responsible for metabolizing isoleucine in *F. sacchari*.

In the medium with leucine as the sole carbon source, reductions in colony size of 57.20% in Δ*FsACAD-2*, 4.1% in Δ*FsACAD-10* and Δ*FsACAD-12*, and 2.5% in Δ*FsACAD-9* were observed. The remaining *FsACAD* mutants had colony sizes similar to those of the wild-type strain (Figure 8 and Figure S4, Table S3). These results demonstrate that FsACAD-2 is the dominant enzyme for the breakdown of leucine as a carbon source in *F. sacchari*.

In medium with tryptophan as the sole carbon source, 7.5%, 6.2%, and 3.6% reductions in colony size were observed in Δ*FsACAD-12*, Δ*FsACAD-2*, and Δ*FsACAD-14*. The remaining *FsACAD* mutants had colony sizes similar to those of the wild-type strain (Figure 8 and Figure S4, Table S3). However, the marginal contribution of FsACAD-12, FsACAD-2, and FsACAD-14 to tryptophan breakdown does not seem sufficient to ensure the vigorous growth of the wild-type strain in the medium with tryptophan as the sole carbon source. It may well be that other enzymes are involved in catalyzing the degradation of tryptophan in *F. sacchari*.

### 2.9. Contribution of FsACADs to Virulence

**Figure 9:**
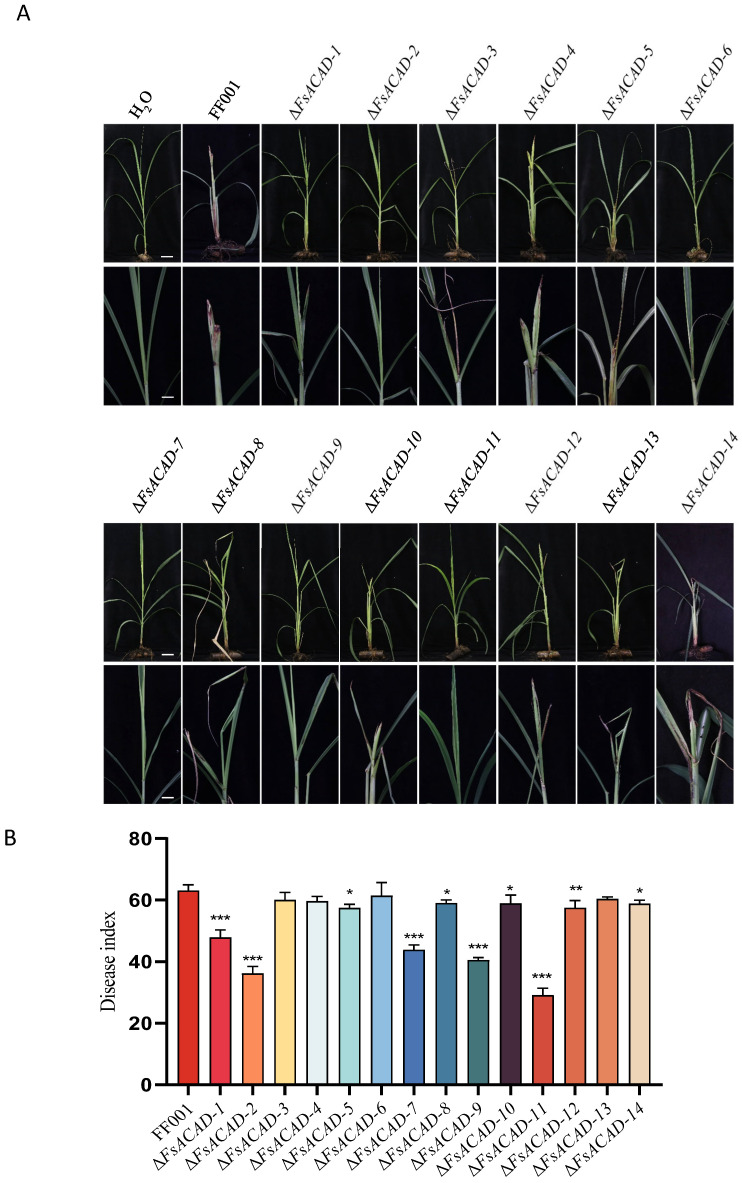
Symptoms and disease index values of mutants of *Fusarium sacchari* acyl-CoA dehydrogenase genes on sugarcane plants. (**A**) Symptoms on sugarcane plants 14 days post-inoculation. The upper panels are overviews of the whole plant, and the lower panels show a close-up of the shoots. The inoculation was performed by injecting 0.5 mL of conidia suspension (10^5^ conidia/mL) into the shoot tissue close to the meristem. Scale bar in the panel represents 15 cm and scale bar in the lower represents 5 cm. (**B**) Disease index values of the inoculated plants. The inoculations were repeated three times, with 15–20 plants for each. * indicates *p* < 0.05; ** indicates *p* < 0.01; *** indicates *p* < 0.001.

Δ*FsACAD* mutants of whole sugarcane plants were assayed for virulence and disease severity (Figure 9A). Disease index (DI) values ranging from 69.57% to 96.15% were recorded for the tested strains and 0% for the mock group. The symptoms induced by Δ*FsACAD-3*, Δ*FsACAD-4*, Δ*FsACAD-6*, and Δ*FsACAD-13* were most severe, with disease index (DI) values of 60 to 61, similar to the wild-type FF001 (DI = 63). Mutants Δ*FsACAD-5*, Δ*FsACAD-8*, Δ*FsACAD-10*, Δ*FsACAD-12*, and Δ*FsACAD-14* exhibited less severe symptoms with DIs of 57–59; Δ*FsACAD-1*, Δ*FsACAD-7*, and Δ*FsACAD-9* displayed mild symptoms with DIs of 40–47. The most attenuated mutants were Δ*FsACAD-11* and Δ*FsACAD-2*, with DIs of 29 and 36, followed by Δ*FsACAD-9*, Δ*FsACAD-7*, and Δ*FsACAD-1*, with DIs of 40, 44, and 48 (Figure 9B).

### 2.10. Phenotype of FsACAD-2/FsACAD-11 Double Knockout Mutants

Since a significant reduction in the disease index was observed in the Δ*FsACAD-2* and Δ*FsACAD-11* mutants, a double knockout of *FsACAD-2* and *FsACAD-11* (Δ*FsACAD-2/-11*) was consequently generated to address whether these two genes exert a compound effect on the growth characteristics and virulence of *F. sacchari* in sugarcane.

The growth rate of Δ*FsACAD-2/-11* consistently fell between those of Δ*FsACAD-2* and Δ*FsACAD-11* across various media. There was a similar level of sporulation on PDA, MM, and CM plates to Δ*FsACAD-2* (Figure S5). The Δ*FsACAD-2/-11* mutant exhibited synergic sensitivity to 0.5M NaCl but with similar responses as for the single-gene mutants Δ*FsACAD-2* and Δ*FsACAD-11* to other stresses (Figure S6).

**Figure 10:**
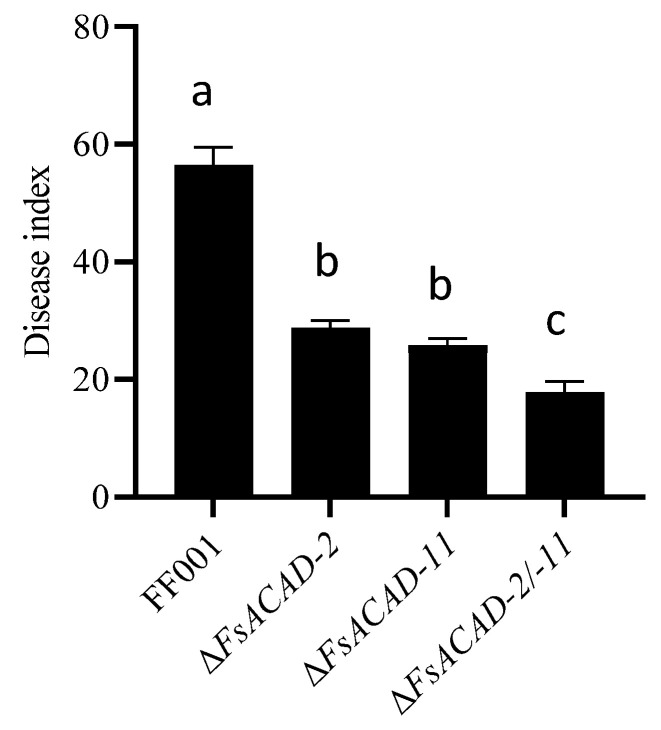
Comparison of virulence between the single and double mutants of *Fusarium sacchari* acyl-CoA dehydrogenase genes *FsACAD-2* and *FsACAD-11*. The inoculations were repeated three times, with 15–20 plants for each. Same letters indicate no significant differences (*p* < 0.05).

Δ*FsACAD-2/-11* exhibited similar growth rates in different fatty acid media to the single-deletion mutants of Δ*FsACAD-2* and Δ*FsACAD-11*, with no significant superposition effect (Figure S7). Δ*FsACAD-2/-11* has a similar amino acid utilization ability to Δ*FsACAD-2* (Figure S8). However, the virulence of Δ*FsACAD-2/-11* was markedly reduced (DI = 18) compared with the single-gene knockout mutants (Figure 10).

## 3. Discussion

The β-oxidation pathway exists in bacteria, fungi, animals, and plants [10,28,29,30,31]. In recent years, the expansion of genomic data and the growing interest in acyl-CoA dehydrogenases have led to the identification and investigation of additional ACAD family members, including IVD, SCAD (short chain-specific acyl-CoA dehydrogenase), and GCD [6,32,33]. While detailed studies on the functions of all 11 members of the ACAD family have been conducted in humans [24,34], few reports are available in other organisms. To the best of our knowledge, this study is the first to identify the functions of all ACADs at the whole-genome scale in a filamentous fungus.

An organism may diversify its ACAD proteins to adapt to the environment by utilizing as many fatty acids as possible. For example, there are 11 ACAD members in humans, and each ACAD may have a distinct function [35,36,37,38,39,40,41]. Of these ACADs, seven types of domains (ACAD superfamily, ACAD, Acyl-CoA_dh_N superfamily, CaiA, Cyt-b5, GCD, and SCAD_SBCAD) were identified [42]. It has been reported that ACAD domains are associated with specificity or efficiency in catalyzing different substrates [43]. Despite significant variation in their primary sequences, many ACADs have similar tertiary structures [35,36,37,38,39,40,41]. This structural similarity may guarantee the redundancy of ACADs to catalyze a vast range of substrates while maintaining adequate preference for specific substrates. As seen in Figure 1, FsACAD proteins in *F. sacchari* are rich in terms of domain types, yet all share a similar tertiary structure (Figure S1).

We noted that the deletion of *FsACAD-2* or *FsACAD-11* resulted in a drastic reduction in the conidiation level in all tested media (PDA, MM, and CM) (Figure 6). In this regard, it was reported that ACAD-2 deficiency leads to the accumulation of various derivative organic acids, such as isovaleric acid, 3-hydroxyisovaleric acid, isovaleryl (C5)-carnitine, and isovaleryl glycine, and ACAD-11 deficiency results in the accumulation of the dicarboxylates glutaric acid, 3-hydroxy-glutaric acid, and glutaconic acid in human patients [44]. The accumulation of these substances could lead to organic acidosis and even trigger the occurrence of cell apoptosis [44,45,46]. Thus, abnormal accumulation of organic acids in the cells may be a reason for the lowered sporulation in the *F. sacchari* mutants.

In terms of utilization efficiency, short-chain fatty acids were preferred over long-chain fatty acids in *F. sacchari* (Figure 7). As judged by mutant performance, *FsACAD-1* was found to be essential for utilization of myristic acid and linoleic acid, *FsACAD-2* for isovaleric acid, *FsACAD-3* for capric acid, *FsACAD-7* for erucic acid, *FsACAD-12* for hexanoic acid and oleic acid, and *FsACAD-13* for octanoic acid, as deletion of the genes encoding these enzymes significantly retarded radial growth of the fungus. Unexpectedly, no significant impact on the utilization of lauric acid was seen in any of the *FsACAD* mutants, suggesting that all of the FsACADs do not participate in the breakdown of lauric acid (Figure 7).

Differences in the efficiency of amino acid catalyzation were profound among the FsACADs. *FsACAD-2* was found to be essential in the utilization of leucine, valine, and tryptophan, and *FsACAD-12* in the utilization of isoleucine, valine, and tryptophan (Figure 8). Thus, FsACAD-2 and FsACAD-12 are considered the primary enzymes in the metabolism of amino acids for growth in *F. sacchari*.

As a parasitic pathogen, nutrient acquisition is vital for *F. sacchari* when it colonizes a sugarcane plant. Indeed, most *FsACADs* were upregulated during the infection of sugarcane plants (Figure 3), suggesting they may be involved in this process. Plant assays revealed that 10 out of the 14 *FsACAD*-deletion mutants were attenuated in virulence, of which Δ*FsACAD-2* and Δ*FsACAD-11* exhibited the lowest disease index values (Figure 9). Unlike Δ*FsACAD-2*, Δ*FsACAD-11* did not seem to have a significant impact on fatty and amino acid utilization. We suspect that *FsACAD-11* may have a different function from *FsACAD-2* in the metabolic pathway. Thus, we then opted to generate a *FsACAD-2* and *FsACAD-11* double mutant (Δ*FsACAD-2/11*) and compared it with the single mutants Δ*FsACAD-2* and Δ*FsACAD-11*. Δ*FsACAD-2/11* did not exhibit a more severe decrease in hyphal growth, sporulation, or stress resistance compared with the single-gene mutants Δ*FsACAD-2* and Δ*FsACAD-11* (Figures S7 and S8). However, Δ*FsACAD-2/11* showed a significant reduction in virulence (Figure 10). The additional attenuation in virulence in the double mutant may likely stem from the buildup of intermediate metabolites or hydrogen peroxide that are cytotoxic and compromise cellular health, as reported for the rice blast fungus [47]. Taking these results together, the current study has unveiled the biological functions of each member of the ACAD family in *F. sacchari* and identified potentially essential ACAD candidates as targets for PBD control. The insights gained may also serve as a reference for other phytopathogenic fungi.

## 4. Materials and Methods

### 4.1. Fungal Strains and Their Culture Conditions

*F. sacchari* wild-type strain FF001 was isolated from a sugarcane leaf showing Pokkah beong symptoms in Fusui County of Guangxi Province, China. This strain was used to generate *FsACAD* knockout mutants (Table S4). The fungal strain was grown on solid potato dextrose agar (PDA) in the dark at 28 °C and transferred to a fresh plate every 7 days to maintain its vigor. PDA, complete medium (CM) [48], and minimal medium (MM) [48] were used to characterize the growth rate and sporulation of the fungal strains. To investigate the dry weight of mycelium, fresh spores (10^5^ spores) of strains were inoculated into 150 mL of PDW, liquid MM, and CM at 28 °C in a rotary shaker at 180 rpm for 72 h. The mycelium was collected by filtering through a Mirachoth filter (Merck & Co., Inc., Darmstadt, Germany) and dehydrated in a 37 °C oven until a constant weight was achieved, followed by weighing the mycelium.

### 4.2. Bioinformatics Analysis

Whole-genome sequencing and annotation of *F. sacchari* FF001 were performed by BioMarker Technologies Company (Beijing, China). The *FsACAD* genes were screened against the whole-genome sequence of *F. sacchari* FF001. The *FsACAD* gene structure domains were predicted using NCBI Batch_CDD (https://www.ncbi.nlm.nih.gov/Structure/bwrpsb/bwrpsb.cgi (accessed on 22 December 2024)) and displayed using TBtools v2.149 [27]. The de novo predictions of the tertiary structure were performed using I-TASSER v5.1 (https://zhanggroup.org/I-TASSER/ (accessed on14 December 2023)). The maximum likelihood method with 5000 bootstraps was used to construct the phylogenetic tree regarding the LG model. The presentation of the tree was optimized by using iTOL (https://itol.embl.de/ (accessed on 25 December 2024)). Reference sequences employed for the construction of the phylogenetic tree were sourced from the NCBI protein database. To obtain the protein sequences of acyl-CoA dehydrogenase (ACAD) from specific filamentous fungi, FsACAD proteins were used to conduct species-specific BLASTP (https://blast.ncbi.nlm.nih.gov/Blast.cgi (accessed on 24 December 2024)) analysis.

### 4.3. Gene Expression Profiling

To investigate the expression profiles of *FsACADs* during fungal infection of sugarcane, the wild-type strain FF001 was cultured in liquid CM at 28 °C in a rotary shaker at 180 rpm for 3 days. The mycelium was collected by filtering through a Mirachoth filter (Merck & Co., Inc., Darmstadt, Germany). A small patch of fungal mycelium was placed on the wound site (a cut using scissors) of the detached young sugarcane leaf piece (7 cm × 3 cm). A total of 20 pieces of sugarcane leaf were used. The inoculated leaf pieces were kept in a container with wet cotton to maintain high humidity at 26 °C for up to 3 days. After washing away the mycelium from the leaf surface, chlorotic spots around the inoculation sites were collected and stored at −80 °C for total RNA extraction. Three biological replicates were used.

To monitor the expression of *FsACADs* in response to fatty acid induction, fresh FF001 spores (10^8^ spores) were inoculated into 150 mL of sucrose-free liquid MM containing 10 mM fatty acid and 0.1% of NP40 (Sigma-Aldrich Co., St Louis, MO, USA) and incubated at 28 °C for 24 h. The fungal mass was harvested by centrifugation and stored at −80 °C for total RNA extraction. Three biological replicates were used.

Total RNA was extracted from the sugarcane leaf tissues or fungal mass by using the FastPure Universal Plant Total RNA Isolation Kit (Nanjing Vazyme Biotech Co., Ltd., Nanjing, China). Reverse transcription of RNA into cDNA was performed using TransScript^®^ Uni All-in-One First-Strand cDNA Synthesis SuperMix (TransGen Biotech Co., Ltd., Beijing, China).

The expression levels of the *FsACAD* genes were analyzed using fluorescence quantitative PCR with PerfectStart^®^ Green qPCR SuperMix (TransGen Biotech Co., Ltd., Beijing, China), with the beta-actin gene as a reference. Primers specific to these genes are listed in Supplementary Table S5. The fluorescence quantitative PCR program was implemented as follows: pre-denaturation at 95 °C for 5 min; 45 cycles of 95 °C for 15 s, 58 °C for 15 s, 72 °C for 15 s, and the detection of fluorescent signals; melting at 95 °C for 10 s; 65 °C for 60 s; 97 °C for 1 s; and cooling at 37 °C for 30 s.

### 4.4. Construction of FsACAD-Deletion Mutants

Fungal genomic DNA was extracted from the mycelium following the method described by Amir et al. [49]. *FsACAD*-deletion mutants were constructed using a homologous recombination strategy following the protocol of Lan et al. [50]. Briefly, the approximately 1 kb upstream and 1 kb downstream flanking fragments of a *FsACAD* gene were amplified using the primers FsACAD-LF/LR and FsACAD-RF/RR (Figure S9, Table S5), respectively. Hygromycin resistance gene (*HphR*) cassette fragments were amplified using the primers HphF/R. The flanking fragments and *HphR* fragments were ligated via overlapping PCR. The homologous recombination fragments were transformed into *F. sacchari* FF001 protoplasts prepared as described by Chen et al. [51]. Mutants grown on solid CM with 100 μg/mL hygromycin [52] were selected for further identification using PCR with primer pairs FsACAD-LF/Hyg-F0, FsACAD-RR/Hyg-R0, and FsACAD-F/FsACAD-R (Table S5) to ensure the target *FsACAD* gene had been replaced with *HphR*. To construct double knockout mutants, geneticin (G418R, 75 μg/mL) was used as the second selection marker [30]. The mutants were purified to homogeneity by single spore isolation.

### 4.5. Stress Assay

For stress sensitivity assays, fungal strains were inoculated onto the CM plates with stressors NaCl, sorbitol, Congo red, SDS, or H_2_O_2_ at designated concentrations [26,52]. The plates were kept in dark at 28 °C for up to 5 days. The inhibition rate is calculated based on the following formula:

$$Inhibition\;ration\;\left(\%\right)=\frac{Diameter\;of\;control-Diameter\;of\;treatment}{Diameter\;of\;control-Diameter\;of\;Inoculate\;blocks}\times 100\%$$

The experiments were performed using three biological replicates.

### 4.6. Assays for Utilization of Fatty and Amino Acids as the Sole Carbon Source

To assay the utilization efficiency of fatty and amino acids as the sole carbon source, fungal strains were inoculated onto sucrose-deficient solid MM containing 10 mM of the fatty acid or amino acid of interest [26]. Regular MM plates with sucrose as the sole carbon source were used as the control. Colony sizes were measured 5 days post-inoculation.

### 4.7. Virulence Assays and Pathogenicity Analysis

The sugarcane variety ZZ9 at the sixth–seventh leaf stage was used to assess the pathogenicity of *FsACAD* gene deletion mutants. A volume of 100 μL of freshly prepared fungal conidia suspension (10^5^ conidia/mL) was injected into the shoot tissue around the meristem point of the plant stalk. Each treatment contained 10 plants, and there were three replicates for each treatment. Sterile water was used as a mock. Disease symptoms were recorded after 14 days post-inoculation. Disease severity was assessed based on the formula proposed by Wang et al. [53,54]:

$$DI=\frac{\sum (NEDSL\times VEDG)}{TNP\times 5}\times 100$$

where DI is the disease index, NEDSL is the number of each disease severity level, VEDG is the value of each disease grade, and TNP is the total number of tested plants.

### 4.8. Data Analysis

Statistical analysis was conducted using IBM SPSS Statistics 22 (SPSS Inc., Chicago, IL, USA). One-way analysis of variance (ANOVA) was applied, considering the experimental results as the dependent variable and the different strains as the independent variable. Duncan’s test (*p* = 0.01) was utilized to identify significant differences between treatments. Data are expressed as mean ± standard deviation. For graphical representations, GraphPad Prism 8 (GraphPad Software, San Diego, CA, USA) was employed.

## 5. Conclusions

Fourteen *FsACAD* genes that are likely involved in fatty acid β-oxidation were identified from the sugarcane Pokkah boeng disease pathogen *F. sacchari*. The functions of these genes were investigated by generation of gene deletion mutants and characterization of mutant performance under various conditions. While most of the *FsACAD* genes responded to fatty acids in terms of expression levels, there were profound variations in the impact on growth and sporulation, in the efficiency of fungal utilization of fatty acids or amino acids, and in the regulation of pathogenicity. Of the *FsACAD* genes, *FsACAD-2* and *FsACAD-11* were the most important for pathogenicity. These two genes and their encoded proteins could serve as potential targets for PBD control.

## Figures and Tables

**Table 1:** Characteristics of *Acyl-CoA dehydrogenase* gene family in *Fusarium sacchari*.

Gene	Accession No.	Chromosome ^a^	Protein Size (aa)	Subcellular Localization	Annotation
*FsACAD-1*	PP466006	Chrom5(+)	381	mitochondria	Acyl-CoA dehydrogenase medium-chain specific mitochondrial precursor
*FsACAD-2*	PP466007	Chrom6(−)	427	mitochondria	Isovaleryl-CoA dehydrogenase
*FsACAD-3*	PP466008	Chrom3(+)	439	mitochondria	Acyl dehydrogenase medium-chain specific mitochondrial precursor
*FsACAD-4*	PP466009	Chrom3(+)	426	mitochondria	Acyl-CoA dehydrogenase medium-chain specific mitochondrial precursor
*FsACAD-5*	PP466010	Chrom4(+)	638	mitochondria	Acyl-CoA dehydrogenase family member 11
*FsACAD-6*	PP466011	Chrom4(+)	548	mitochondria	Acyl-CoA dehydrogenase
*FsACAD-7*	PP466012	Chrom4(−)	469	mitochondria	Long-chain specific acyl-CoA dehydrogenase
*FsACAD-8*	PP466013	Chrom4(−)	441	mitochondria	Long-chain specific acyl-CoA dehydrogenase
*FsACAD-9*	PP466014	Chrom7(−)	514	mitochondria	Very long-chain specific acyl-CoA dehydrogenase
*FsACAD-10*	PP466015	Chrom7(+)	438	mitochondria	Acyl-CoA dehydrogenase related to the alkylation response protein AidB
*FsACAD-11*	PP466016	Chrom7(+)	480	mitochondria	Glutaryl-CoA dehydrogenase
*FsACAD-12*	PP466017	Chrom7(+)	446	mitochondria	Short/branched chain-specific acyl-CoA dehydrogenase
*FsACAD-13*	PP466018	Chrom8(+)	443	mitochondria	Acyl-CoA dehydrogenase fadE12
*FsACAD-14*	PP466019	Chrom2(+)	426	mitochondria	Acyl-CoA dehydrogenase

^a^ coding strain, + = positive DNA strand and − = negative DNA strand.

**Table 2:** Colony size of mutants of *Fusarium sacchari* acyl-CoA dehydrogenase genes in different media *.

Strain	Average Colony Size (cm^2^)		
	PDA	MM	CM
FF001	36.14 ± 0.312 ^bc^	48.81 ± 0.358 ^c^	46.37 ± 0.352 ^a^
Δ*FsACAD-1*	33.70 ± 1.372 ^ef^	47.58 ± 0.352 ^d^	46.37 ± 0.352 ^a^
Δ*FsACAD-2*	29.39 ± 0.283 ^g^	45.96 ± 0.605 ^ef^	32.68 ± 0.883 ^i^
Δ*FsACAD-3*	32.68 ± 0.505 ^f^	43.59 ± 0.585 ^g^	44.58 ± 1.484 ^bc^
Δ*FsACAD-4*	35.43 ± 0.300 ^cd^	47.58 ± 0.352 ^d^	43.20 ± 0.335 ^de^
Δ*FsACAD-5*	38.85 ± 0.323 ^a^	46.77 ± 0.699 ^de^	45.57 ± 0.687 ^ab^
Δ*FsACAD-6*	33.35 ± 0.300 ^ef^	46.57 ± 0.605 ^e^	45.37 ± 0.595 ^ab^
Δ*FsACAD-7*	24.34 ± 0.254 ^h^	50.06 ± 0.364 ^b^	35.26 ± 0.525 ^h^
Δ*FsACAD-8*	33.87 ± 0.789 ^ef^	45.56 ± 0.346 ^f^	42.24 ± 0.335 ^e^
Δ*FsACAD-9*	34.39 ± 0.300 ^de^	47.58 ± 0.352 ^d^	43.99 ± 1.224 ^cd^
Δ*FsACAD-10*	36.14 ± 0.312 ^bc^	46.37 ± 0.352 ^ef^	42.62 ± 0.335 ^e^
Δ*FsACAD-11*	33.19 ± 1.357 ^ef^	45.96 ± 0.605 ^ef^	38.85 ± 0.641 ^g^
Δ*FsACAD-12*	33.01 ± 0.294 ^f^	47.58 ± 0.352 ^d^	42.62 ± 0.335 ^e^
Δ*FsACAD-13*	33.35 ± 0.300 ^ef^	47.58 ± 0.352 ^d^	45.56 ± 0.346 ^ab^
Δ*FsACAD-14*	37.22 ± 0.824 ^b^	51.11 ± 0.364 ^a^	40.16 ± 0.976 ^f^

* Values represent the mean and standard error of multiple observations obtained from three replicates. Columns with identical letters have no statistically significant differences, as determined by Duncan’s test (*p* < 0.05). Each mutant strain was inoculated on three plates for each medium. For each strain and medium within each treatment, means followed by the same letter are not significantly different.

**Table 3:** Spore yield of mutants of *Fusarium sacchari* acyl-CoA dehydrogenase genes in different media *.

Strains	PDA Medium		MM		CM	
	Average (Spore/cm^2^)	Fold Change	Average (Spore/cm^2^)	Fold Change	Average (Spore/cm^2^)	Fold Change
FF001	3.95 × 10^6^ ± 0.69 × 10^6^	1.00 ^bcd^	3.69 × 10^6^ ± 0.59 × 10^6^	1.00 ^d^	5.81 × 10^6^ ± 1.52 × 10^6^	1.00 ^a^
Δ*FsACAD-1*	3.57 × 10^6^ ± 0.90 × 10^6^	0.90 ^bcd^	7.38 × 10^6^ ± 1.43 × 10^6^	2.00 ^b^	6.30 × 10^6^ ± 0.58 × 10^6^	1.09 ^a^
Δ*FsACAD-2*	0.60 × 10^6^ ± 0.06 × 10^6^	0.15 ^e^	1.59 × 10^6^ ± 0.30 × 10^6^	0.43 ^e^	1.42 × 10^6^ ± 0.14 × 10^6^	0.24 ^d^
Δ*FsACAD-3*	2.80 × 10^6^ ± 1.29 × 10^6^	0.71 ^cd^	4.67 × 10^6^ ± 0.79 × 10^6^	1.27 ^d^	4.07 × 10^6^ ± 0.37 × 10^6^	0.70 ^bc^
Δ*FsACAD-4*	3.10 × 10^6^ ± 1.50 × 10^6^	0.78 ^cd^	6.81 × 10^6^ ± 0.86 × 10^6^	1.85 ^b^	4.14 × 10^6^ ± 0.37 × 10^6^	0.71 ^bc^
Δ*FsACAD-5*	6.96 × 10^6^ ± 0.62 × 10^6^	1.76 ^a^	7.27 × 10^6^ ± 0.90 × 10^6^	1.97 ^b^	5.50 × 10^6^ ± 1.14 × 10^6^	0.95 ^ab^
Δ*FsACAD-6*	3.87 × 10^6^ ± 0.66 × 10^6^	0.98 ^bcd^	4.35 × 10^6^ ± 0.32 × 10^6^	1.18 ^d^	4.09 × 10^6^ ± 0.05 × 10^6^	0.70 ^bc^
Δ*FsACAD-7*	2.28 × 10^6^ ± 0.45 × 10^6^	0.58 ^de^	10.11 × 10^6^ ± 0.70 × 10^6^	2.74 ^a^	3.55 × 10^6^ ± 0.53 × 10^6^	0.61 ^c^
Δ*FsACAD-8*	3.95 × 10^6^ ± 0.42 × 10^6^	1.00 ^bcd^	4.57 × 10^6^ ± 0.24 × 10^6^	1.24 ^d^	5.20 × 10^6^ ± 0.29 × 10^6^	0.9 ^ab^
Δ*FsACAD-9*	5.04 × 10^6^ ± 0.58 × 10^6^	1.28 ^b^	4.51 × 10^6^ ± 0.15 × 10^6^	1.22 ^d^	3.11 × 10^6^ ± 0.31 × 10^6^	0.54 ^c^
Δ*FsACAD-10*	4.39 × 10^6^ ± 1.09 × 10^6^	1.11 ^bc^	6.35 × 10^6^ ± 0.41 × 10^6^	1.72 ^bc^	3.37 × 10^6^ ± 0.29 × 10^6^	0.58 ^c^
Δ*FsACAD-11*	0.73 × 10^6^ ± 0.03 × 10^6^	0.19 ^e^	1.56 × 10^6^ ± 0.19 × 10^6^	0.42 ^e^	0.77 × 10^6^ ± 0.03 × 10^6^	0.13 ^d^
Δ*FsACAD-12*	2.70 × 10^6^ ± 0.43 × 10^6^	0.69 ^cd^	4.99 × 10^6^ ± 0.63 × 10^6^	1.35 ^cd^	5.51 × 10^6^ ± 0.15 × 10^6^	0.95 ^ab^
Δ*FsACAD-13*	3.63 × 10^6^ ± 0.57 × 10^6^	0.92 ^bcd^	1.98 × 10^6^ ± 0.53 × 10^6^	0.54 ^e^	3.65 × 10^6^ ± 0.53 × 10^6^	0.63 ^c^
Δ*FsACAD-14*	5.42 × 10^6^ ± 0.31 × 10^6^	1.37 ^ab^	4.63 × 10^6^ ± 0.46 × 10^6^	1.26 ^d^	5.53 × 10^6^ ± 0.32 × 10^6^	0.95 ^ab^

* The spore production per unit area of *FsACAD* mutants relative to FF001. The values represent the mean and standard error of multiple observations obtained from three replicates. Columns with identical letters have no statistically significant differences, as determined by Duncan’s test (*p* < 0.05).

## Data Availability

The data presented in this study are available in the insert article or Supplementary Materials here.

## Supplementary Materials

The following supporting information can be downloaded at https://www.mdpi.com/article/10.3390/ijms26030973/s1.
