# Supplementary material for: Genome-Wide Identification and Functional Characterization of the Acyl-CoA Dehydrogenase (ACAD) Family in Fusarium sacchari
Source: Int J Mol Sci. 2025 Jan 24;26(3):973. doi: 10.3390/ijms26030973 (PMC11817166; doi:10.3390/ijms26030973)
Supplement: Supplementary file 1 [file ijms-26-00973-s001.zip › ijms-3392871-supplementary.pdf]

Table S1. Mycelial dry weight of *Fusarium sacchari* acyl-CoA dehydrogenase mutants in different media*

Strains	Dry weight (g)		
	PDW	MM	CM
FF001	0.236±0.009c	0.656±0.125bcde	0.754±0.008efg
Δ*FsACAD-1*	0.269±0.002a	0.664±0.008bcde	0.773±0.01cd
Δ*FsACAD-2*	0.188±0.007hi	0.764±0.017a	0.868±0.023a
Δ*FsACAD-3*	0.218±0.002de	0.610±0.018g	0.706±0.006i
Δ*FsACAD-4*	0.209±0.005ef	0.630±0.009efg	0.691±0.005j
Δ*FsACAD-5*	0.184±0.005i	0.677±0.032bc	0.726±0.004h
Δ*FsACAD-6*	0.223±0.001d	0.683±0.006b	0.787±0.003c
Δ*FsACAD-7*	0.271±0.011a	0.621±0.022fg	0.748±0.005fg
Δ*FsACAD-8*	0.216±0.003def	0.672±0.016bcd	0.760±0.006def
Δ*FsACAD-9*	0.214±0.007def	0.638±0.02defg	0.761±0.012def
Δ*FsACAD-10*	0.248±0.006b	0.648±0.019cdef	0.748±0.011fg
Δ*FsACAD-11*	0.237±0.001c	0.748±0.018a	0.836±0.009b
Δ*FsACAD-12*	0.196±0.012gh	0.661±0.012bcde	0.773±0.003cd
Δ*FsACAD-13*	0.207±0.074ef	0.641±0.015defg	0.768±0.004de
Δ*FsACAD-14*	0.206±0.001fg	0.621±0.028fg	0.743±0.003g

* Mycelial dry weight was measured 3 days after cultivation. Values represent the mean and standard error of multiple observations obtained from three replicates. Columns sharing identical letters indicate no statistically significant difference as determined by Duncan's test (p < 0.05).

Table S2. Colony size of the *Fusarium sacchari* acyl-CoA dehydrogenase mutants on solid media with different fatty acid as sole carbon source*

Strain	Sucrose	Isovaleric acid	Valeric acid	Hexanoic acid	Erucic acid	Octanoic acid	Oleic acid	Myristic acid	Linoleic acid	Lauric acid	Capric acid
FF001	50.06±0.364def	46.77±0.346a	37.03±0.312bcd	35.26±0c	31.84±0.577cde	27.97±1.424bcd	27.50±0.708bcd	27.03±0.266abcd	23.36±2.238ab	16.02±0.208e	14.86±0.199abcd
Δ*FsACAD-1*	48.81±0.358f	46.37±0.352a	37.39±0.545abc	36.15±1.547bc	32.50±0.289cde	27.81±0.465bcd	22.15±1.133g	10.37±0.162j	8.64±0.398g	17.36±0.740bcde	10.21±0.849e
Δ*FsACAD-2*	53.03±0.375a	35.26±0.525f	37.57±0.318ab	38.12±0.629a	33.19±0.883bcd	28.12±0.266abcd	27.19±0.707cd	19.39±1.463gh	14.19±1.433f	19.79±1.781abc	4.37±0.534f
Δ*FsACAD-3*	51.74±0.370b	40.91±0.323cd	37.04±0.826bcd	35.62±1.218c	34.39±0.795b	26.27±0.691e	28.91±0.733b	19.12±0.970h	16.04±1.150ef	20.98±1.314a	16.28±0.739ab
Δ*FsACAD-4*	50.06±0.727def	42.43±0.580b	38.12±1.138a	35.96±0.312bc	32.67±0.505cde	27.96±0.537bcd	26.58±0.946de	26.73±0.697cd	16.91±2.067ef	20.42±3.817a	16.02±2.040ab
Δ*FsACAD-5*	50.20±1.078cde	41.88±0.536bc	35.88±0.355efgh	35.56±0.499c	33.34±0.803bc	26.22±0.700e	26.40±0.920de	26.85±0.292bcd	26.68±0.942a	20.89±1.864a	12.38±0.449bcde
Δ*FsACAD-6*	51.53±0.635bc	42.04±0.335bc	36.32±0.535efg	35.43±0.300c	32.34±0.289cde	28.75±0.475ab	21.66±1.235g	18.73±0.798h	26.74±1.328a	20.57±0.616a	10.52±1.873de
Δ*FsACAD-7*	49.85±0.958ef	42.43±0.999b	35.79±0.918fgh	34.74±1.040c	24.65±1.536g	28.59±0.548abc	28.44±1.097bc	29.08±1.097a	25.08±1.168a	19.05±2.738abcd	6.38±0.130f
Δ*FsACAD-8*	50.56±0.440bcde	37.50±1.046e	36.50±0.306cdefg	37.15±1.243ab	31.83±0.295cde	28.39±0.760abcd	31.05±0.447a	28.92±0.717ab	21.04±1.389bc	18.93±0.382abcd	16.00±0.324ab
Δ*FsACAD-9*	50.90±0.630bcde	42.62±0.670b	36.85±0bcde	35.43±0.606c	32.17±0.500cde	27.65±0.537bcd	24.50±1.539f	25.37±0.260de	20.39±3.324bcd	19.63±0abc	12.85±1.418bcde
Δ*FsACAD-10*	50.48±1.306bcde	38.61±0.454e	35.26±0.525h	36.17±0.696bc	31.56±0.799de	27.24±0.352de	30.93±0.416a	28.27±1.215abc	21.00±1.111bc	19.63±0abc	18.66±1.369a
Δ*FsACAD-11*	51.32±0.364bcd	40.15±0.976d	36.67±0.306bcdef	37.21±0.312ab	37.04±1.360a	27.96±0.537bcd	31.84±0.577a	21.51±0.626f	17.23±0.772def	20.30±1.166a	14.74±2.146abcd
Δ*FsACAD-12*	50.06±0.957def	42.05±1.446bc	35.61±0.300gh	30.19±0d	28.12±0.978f	18.45±0.340f	19.64±1.045h	16.75±0.917i	17.53±1.569def	17.23±0.843cde	10.57±1.514de
Δ*FsACAD-13*	51.53±0.635bc	40.15±0d	36.50±0.306cdefg	36.14±0.810bc	31.17±0.495e	27.50±0.271cd	25.08±1.168ef	24.20±0.756e	24.56±3.227a	20.16±0.611ab	11.25±0.393cde
Δ*FsACAD-14*	50.69±0.364bcde	42.04±0.335bc	36.67±0.306bcdef	35.26±0.525c	31.35±1.604e	29.22±0a	24.20±0.756f	21.20±3.392fg	17.92±2.815cde	16.50±0.208de	15.28±2.381abc

* Colony size (cm^2) of the *FsACAD* mutants on solid media supplemented with different fatty acids for 5 d. The values represent the mean and standard error of multiple observations obtained from three replicates. Columns sharing identical letters indicate no statistically significant difference as determined by Duncan's test ($P < 0.05$). Each mutant strain was inoculated on three plates for each medium. Same letters indicate not significantly different ($p < 0.05$).

Table S3 Colony size of the *Fusarium sacchari* acyl-CoA dehydrogenase mutants on media with different amino acid as sole carbon source*.

Stain	Sucrose (cm²)	Isoleucine (cm²)	Valine (cm²)	Leucine (cm²)	Tryptophan
FF001	50.06±0.364e	50.69±0.364ef	50.48±0.364cd	50.27±0de	43.40±0.676e
Δ*FsACAD-1*	50.69±0.364de	51.53±0.636cd	50.27±0.630d	50.27±0de	45.16±0.6341c
Δ*FsACAD-2*	48.40±0g	46.77±0.350h	37.39±0f	21.51±0.237i	40.72±0g
Δ*FsACAD-3*	51.74±0.370b	51.11±0.730de	50.69±0.364bcd	50.69±0.364d	44.77±0.590cd
Δ*FsACAD-4*	50.90±0cd	51.53±0cd	50.27±0d	45.96±0.601h	44.77±0cd
Δ*FsACAD-5*	50.27±0de	50.06±0.362f	50.48±0.364cd	50.27±0de	43.40±0.335e
Δ*FsACAD-6*	51.53±0bc	52.38±0.371ab	51.32±0.733bc	51.53±0c	45.16±0.341c
Δ*FsACAD-7*	51.53±0.635bc	51.96±0.739bc	50.69±0.364bcd	50.06±0.362de	44.97±0.341c
Δ*FsACAD-8*	50.27±0de	50.48±0.364ef	49.85±0.722d	51.53±0c	44.18±0d
Δ*FsACAD-9*	50.69±0.364de	52.81±0a	51.53±0b	49.02±0.620f	46.57±0.605b
Δ*FsACAD-10*	50.90±0.630cd	52.17±0abc	50.69±0.364bcd	48.19±0.358g	43.20±0.335e
Δ*FsACAD-11*	50.27±0de	52.81±0a	51.53±0.635b	53.24±0.375a	45.36±0c
Δ*FsACAD-12*	49.43±0.358f	40.72±0.565i	35.26±1.055g	48.19±0.358g	40.15±0.565g
Δ*FsACAD-13*	52.38±0.370a	52.60±0.371ab	52.60±0.370a	52.38±0.370b	50.27±0a
Δ*FsACAD-14*	50.27±0.630de	49.02±0.620g	47.17±0.605e	49.64±0.625e	41.85±0.575f

* Colony size (cm²) were measured 5 days post cultivation. The values represent the mean and standard error of multiple observations obtained from three replicates. Columns sharing identical letters indicate no statistically significant difference as determined by Duncan's test (P < 0.05). Each mutant strain was inoculated on three plates for each medium. Same letters indicate not significantly different ($p < 0.05$).

Table S4. Strains used in this study

Strains	Description
Fusarium sacchari FF001	Wild-type strain
Δ*FsACAD-1*	*FsACAD-1* gene deletion mutant of *F. sacchari* FF001
Δ*FsACAD-2*	*FsACAD-2* gene deletion mutant of *F. sacchari* FF001
Δ*FsACAD-3*	*FsACAD-3* gene deletion mutant of *F. sacchari* FF001
Δ*FsACAD-4*	*FsACAD-4* gene deletion mutant of *F. sacchari* FF001
Δ*FsACAD-5*	*FsACAD-5* gene deletion mutant of *F. sacchari* FF001
Δ*FsACAD-6*	*FsACAD-6* gene deletion mutant of *F. sacchari* FF001
Δ*FsACAD-7*	*FsACAD-7* gene deletion mutant of *F. sacchari* FF001
Δ*FsACAD-8*	*FsACAD-8* gene deletion mutant of *F. sacchari* FF001
Δ*FsACAD-9*	*FsACAD-9* gene deletion mutant of *F. sacchari* FF001
Δ*FsACAD-10*	*FsACAD-10* gene deletion mutant of *F. sacchari* FF001
Δ*FsACAD-11*	*FsACAD-11* gene deletion mutant of *F. sacchari* FF001
Δ*FsACAD-12*	*FsACAD-12* gene deletion mutant of *F. sacchari* FF001
Δ*FsACAD-13*	*FsACAD-13* gene deletion mutant of *F. sacchari* FF001
Δ*FsACAD-14*	*FsACAD-14* gene deletion mutant of *F. sacchari* FF001
Δ*FsACAD-2/11*	*FsACAD-2* and *FsACAD-11* genes double deletion mutant of *F. sacchari* FF001

Table S5. Primers used in the study

Primer name	Sequence
HyB F0	GGACGCACTGACGGTGTCGT
HyB R0	CGTCTGGACCGATGGCTGTG
HphF	CGGTACCCGGGGATCCTCTAG
HphR	GCCTGCAGGTCGACAGAAGATG
FsACAD-1L-F	GTTGAGGAGAGCAAGCGAGTAGTGAG
FsACAD-1L-R	ATATCATCTTCTGTCGACCTGCAGGCTTCTCACAATGAGAGGGATTCTCGC
FsACAD-1R-F	TCTTTCTAGAGGATCCCCGGGTACCGTAGAGCGCAGAGTGGCGTGG
FsACAD-1R-R	TTGCTGGCGTGCTTGTTTCG
FsACAD-1F	GCCAAGGAAGCCATCAACAT
FsACAD-1R	CGCTGGTTCAAGGTTAGACTT
FsACAD-2L-F	GGCTTGGTTATAGAAGATGCGACC
FsACAD-2L-R	ATATCATCTTCTGTCGACCTGCAGGCTCCACGGCTGAGATGAATGAAGT
FsACAD-2R-F	TCTTTCTAGAGGATCCCCGGGTACCGCATGTTGCGTGGGTTCACCTATGA
FsACAD-2R-R	GCTCCCGAGGAACAGCCTAACT
FsACAD-2F	CTCTTGCGACAGCTTGCGCTC
FsACAD-2R	GCACACGGCATGAAGCCAAG
FsACAD-3L-F	GCCACGACCTTGATTGAAACG
FsACAD-3L-R	TCCTTCAATATCATCTTCTGGGCTTTGGATGCAGAGACTCTTG
FsACAD-3R-F	ACTCATTTATCAACCGAGATTCTGAGG
FsACAD-3R-R	GTTTAGAGGTAATCCTTCTTATGCAACTTTAACAAAGACATTTCCC
FsACAD-3F	GCCTCCACTCGGAGCAAATC
FsACAD-3R	CTCTTCTTGTCTTGGGCAATGG
FsACAD-4L-F	GCCATCCGTCATTATCCAACCAATA
FsACAD-4L-R	ATATCATCTTCTGTCGACCTGCAGGCGGTGTCGGTAAGAGGACCAATAAG
FsACAD-4R-F	TCTTTCTAGAGGATCCCCGGGTACCGAGCACCTAGGATGCCTATCATT
FsACAD-4R-R	AGTGTCGGTAAAGGTCGAGAAC
FsACAD-4F	GTCTGACACGATGACACCTCA
FsACAD-4R	AGTACCCGAGCCACTACCTTTA
FsACAD-5L-F	TCTATCTCAATGGCAAGCCTCTG
FsACAD-5L-R	ATATCATCTTCTGTCGACCTGCAGGCGCTGCTTTATCACTGTTGGTCTG
FsACAD-5R-F	TCTTTCTAGAGGATCCCCGGGTACCGTTAGTCGTCCAGGCGGATCAC
FsACAD-5R-R	TCAAGACCAATGCGGAGCATATAC
FsACAD-5F	TCTATCTCAATGGCAAGCCTCTG
FsACAD-5R	TCAAGACCAATGCGGAGCATATAC
FsACAD-6L-F	ACTCGTTGGGTTATGCTGAATCAAC
FsACAD-6L-R	ATATCATCTTCTGTCGACCTGCAGGCTGACTTGGTATTGGCGATGATATGAG
FsACAD-6R-F	AGCAGGTGAGGAGCTGCAAGTC
FsACAD-6R-R	TCTTTCTAGAGGATCCCCGGGTACCGAAGCCGGAGTAGAGATCATTTGTATACC
FsACAD-6F	AGCTGTTCGGATGGTTTGTCG
FsACAD-6R	AGGTTTCTTGTCGTCGTGGAGTG
FsACAD-7L-F	ACGGACTCTGAACCGCCTAAT
FsACAD-7L-R	ATATCATCTTCTGTCGACCTGCAGGCTGCGTAGCTCTGGTGGTGAAT
FsACAD-7R-F	TCTTTCTAGAGGATCCCCGGGTACCGATCTGCTTCTAGTTACCCACCAA
FsACAD-7R-R	CGCCATTGACCAAACTCCAA
FsACAD-7F	GAGTTATTGGCACAGCTTCAAGTAG
FsACAD-7R	GTCATCGTGTCTCAGAAGTTCCT

FsACAD-8L-F	TCGAGTTACATACTGCACTGGATTC
FsACAD-8L-R	ATATCATCTTCTGTCGACCTGCAGGCGAGAGCAAGAGCTTGTCAATAGTTG
FsACAD-8R-F	TCTTTCTAGAGGATCCCCGGGTACCGCTCATAAAGCCCGTCTGTTCATAG
FsACAD-8R-R	GGATTCACAGAACGAAGACAAGG
FsACAD-8F	TTCCTAAGTGGCTGTGCAATGG
FsACAD-8R	ACTCGTGCTGGACTTCTTCAATC
FsACAD-9L-F	GTGAGAACTAAGACTGACTGGACTG
FsACAD-9L-R	ATATCATCTTCTGTCGACCTGCAGGCGGAATATTAAGGCGAGACGAGGTAT
FsACAD-9R-F	TCTTTCTAGAGGATCCCCGGGTACCGGCAGTGTTTGAAGGTGGCTATG
FsACAD-9R-R	GCTGAGTTGGCTCCTTGACAT
FsACAD-9F	GCAAGCGAGATAGAGCAGGATG
FsACAD-9R	ACATACGTCTTGGCACAGATAGC
FsACAD-10L-F	CGCTGTAAGGCTTCTATTAGCA
FsACAD-10L-R	ATATCATCTTCTGTCGACCTGCAGGCGTTCCACTAAGGATAAAGTGGTAGT
FsACAD-10R-F	TCTTTCTAGAGGATCCCCGGGTACCGCGATGCTGGAGGCGGCTATA
FsACAD-10R-R	GAATTGGCTGGTAAGCGTGTCA
FsACAD-10F	CGCTGTAAGGCTTCTATTAGCA
FsACAD-10R	GAATTGGCTGGTAAGCGTGTCA
FsACAD-11L-F	GTCAAGGCATGGAAATACCACGAAA
FsACAD-11L-R	ATATCATCTTCTGTCGACCTGCAGGCGGAGATGTGTTATGGATCGGATCAG
FsACAD-11R-F	TCTTTCTAGAGGATCCCCGGGTACCGCCATTGAGGTGCATACCCGTGTTAT
FsACAD-11R-R	TGAGGGCGAATCAGTGTTTAGCG
FsACAD-11F	CTGGGATGCCGAGACGATCATC
FsACAD-11R	GCAGGTAGTCACTAACTGTTCTTCGCC
FsACAD-12L-F	TGAGGCCAGCAGGTAATATACATAC
FsACAD-12L-R	ATATCATCTTCTGTCGACCTGCAGGCAGGAGTGAGGAAGAGTGGTGAC
FsACAD-12R-F	TCTTTCTAGAGGATCCCCGGGTACCGTAGAAAGAGGCAGAGGTACAAATGG
FsACAD-12R-R	CGAGTTTATGGCTCCCGAAGTAT
FsACAD-12F	CCTCGAACAAGATGGTATCTCACA
FsACAD-12R	ATAAGAAGTACGGTCGTGCTGTC
FsACAD-13L-F	GGTGTTGAGAAGGTCATCATCGG
FsACAD-13L-R	ATATCATCTTCTGTCGACCTGCAGGCGATGCCAGATGTCGGTTCTAGTG
FsACAD-13R-F	ATATCATCTTCTGTCGACCTGCAGGCGGAGGCTAGAACAGATGCTTGG
FsACAD-13R-R	GCCGTCTCACCACAGAATAGG
FsACAD-13F	GGTGTTGAGAAGGTCATCATCGG
FsACAD-13R	GCCGTCTCACCACAGAATAGG
FsACAD-14L-F	GCGTACATTGGTGTTGGAGTTG
FsACAD-14L-R	ATATCATCTTCTGTCGACCTGCAGGCAATAAAGTGATGGCAGTGGTTTGAG
FsACAD-14R-F	TCTTTCTAGAGGATCCCCGGGTACCGATATAGCCAATGAGAATGCCATACC
FsACAD-14R-R	CGTGTCTTTGACAATAAGTCAGAAG
FsACAD-14F	GCGTACATTGGTGTTGGAGTTG
FsACAD-14R	CGTGTCTTTGACAATAAGTCAGAAG
Actin-F	CGATTCTGGTGATGGTGTT
Actin-R	ACTCTTCCGTAGCAATGTC
FsACAD-1qF	GGTATCCGAACACTGCGTCTTG
FsACAD-1qR	TTGATTGTCGCTGTCGCCTCA
FsACAD-2qF	GCATGCGAACAACAGCAAAGGCC
FsACAD-2qR	CGTAAACCACAATAACATCGGCG

FsACAD-3qF	CAGTTCTCTCGCTTCCAGGCTACC
FsACAD-3qR	GATGCTCTCGTGAACAAGACCCTC
FsACAD-4qF	ACCTTGATGGTCCTGCTTGTGACTC
FsACAD-4qR	CGTGGTCAACGGGGACTTTGAAG
FsACAD-5qF	TGAGGCAGCGTATTGAACGAGAATC
FsACAD-5qR	CGTCACTACCAGCGTCAACATAGAG
FsACAD-6qF	CCTAAGCCTGAGGCCAATGCTG
FsACAD-6qR	CAGGGTCAGCAAAGGGAATCTGG
FsACAD-7qF	CAGGCCAGAGTTGCTCTGAGCAC
FsACAD-7qR	CGTGTGCGATTCCAACATTGC
FsACAD-8qF	CGAGCGTATGTTCATCTCTGTTGGT
FsACAD-8qR	AGTCTTGTTGAATGCCTTGCGTTGA
FsACAD-9qF	TGACTTGATCGTGGCACAGGAG
FsACAD-9qR	ACCTCGTTGGCAATCTTGTTCTTC
FsACAD-10qF	TGAGGACATCCCTAACCTAGCAGAA
FsACAD-10qR	GGCAATAGAGACTCCACCTTCAACA
FsACAD-11qF	AGCTTGGTCTGTTGGGCGCTAC
FsACAD-11qR	GACCCTCTCCACAGCTCGTGTG
FsACAD-12qF	CCTCTTTCTGTCTCTCTGAGCCTGTCTC
FsACAD-12qR	GGCTTCCTTGGAGTTTGTGATCCAC
FsACAD-13qF	CGAGCAAGACCCAAAGGAGTTCC
FsACAD-13qR	TCTGCATCATCATCGTGGCCTC
FsACAD-14qF	AACGTAGTCGGAACACCTGAGCAAG
FsACAD-14qR	CGATCTTATCTCCGTTGGAGGTGATC

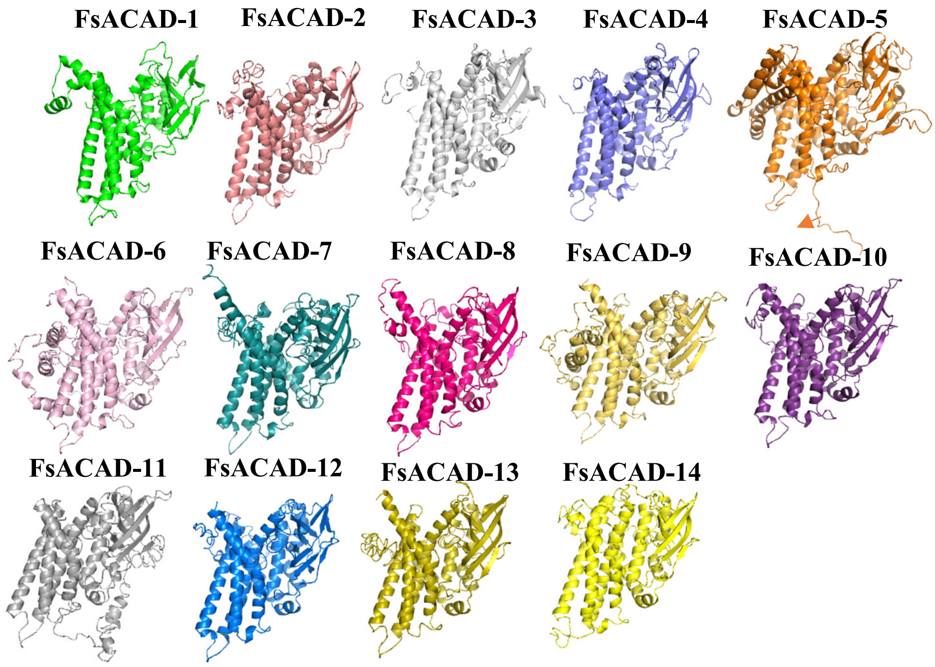

Figure S1. Computer modeling of *Fusarium sacchari* acyl-CoA dehydrogenases. De novo tertiary modeling prediction was conducted using I-TASSER, and the model with the lowest energy was selected and visualized with PyMOL.

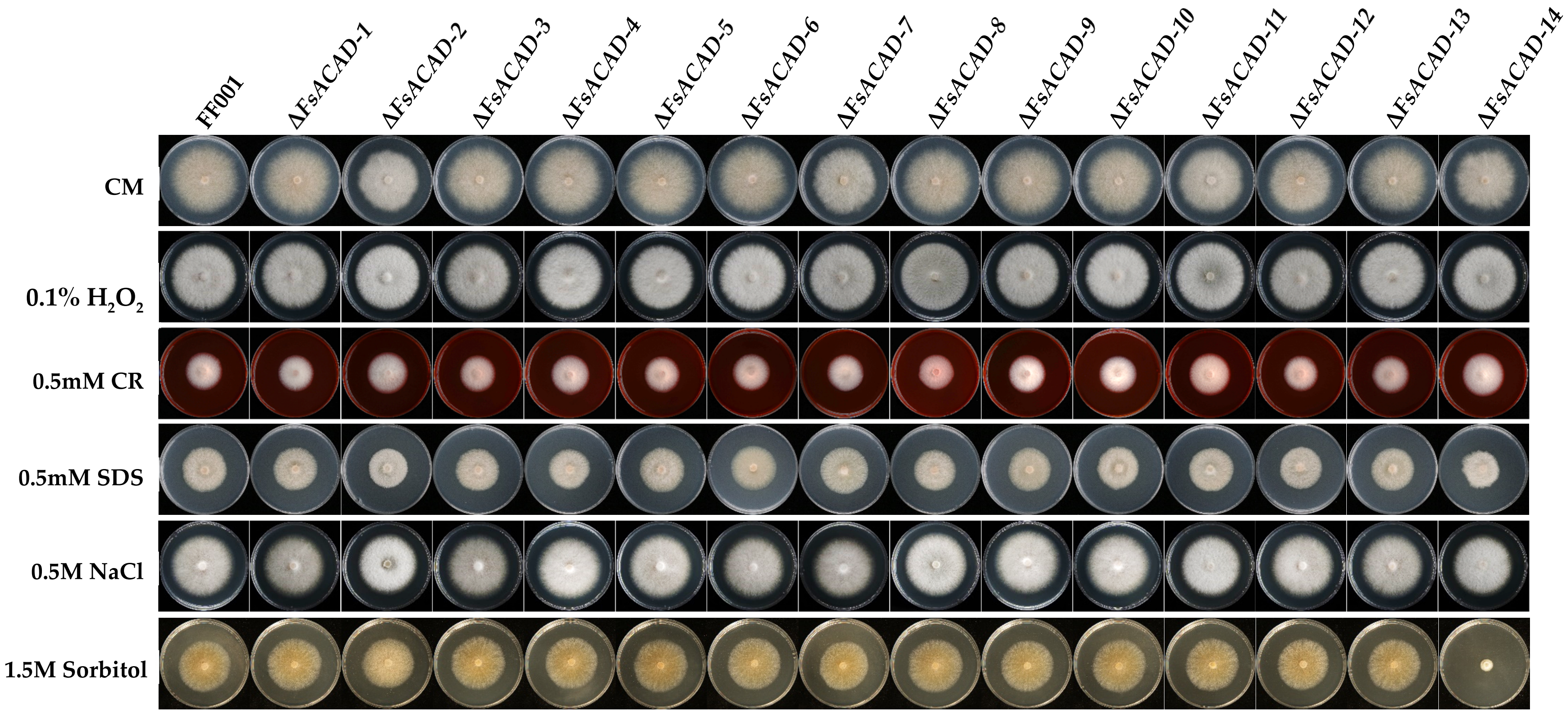

Figure S2. Colonies of *Fusarium sacchari* acyl-CoA dehydrogenases mutants under stress conditions. Inhibition rates of FF001 and *FsACAD* mutant strains on CM medium plates with 0.1% H_2O_2, 0.5mM CR, 0.5mM SDS, 0.5M NaCl, and 1.5M Sorbitol, respectively. The petri dish is 90mm in diameter.

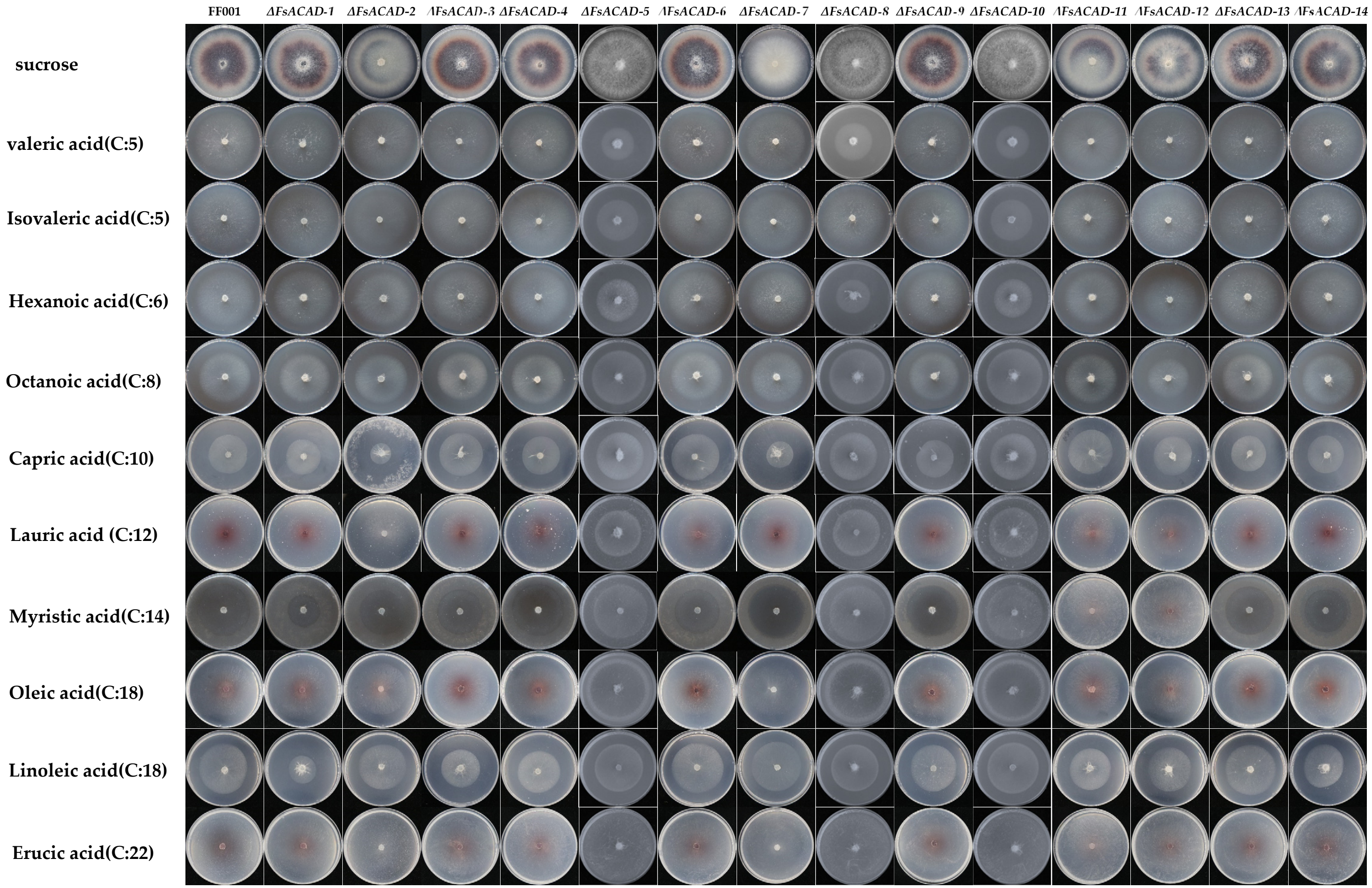

Figure S3. The growth areas of the *Fusarium sacchari* acyl-CoA dehydrogenases (FsACADs) mutants in different carbon sources. The colony growth areas of the *FsACAD* mutants cultured on different lengths of fatty acids as the sole carbon replaced sucrose. The petri dish is 90mm in diameter.

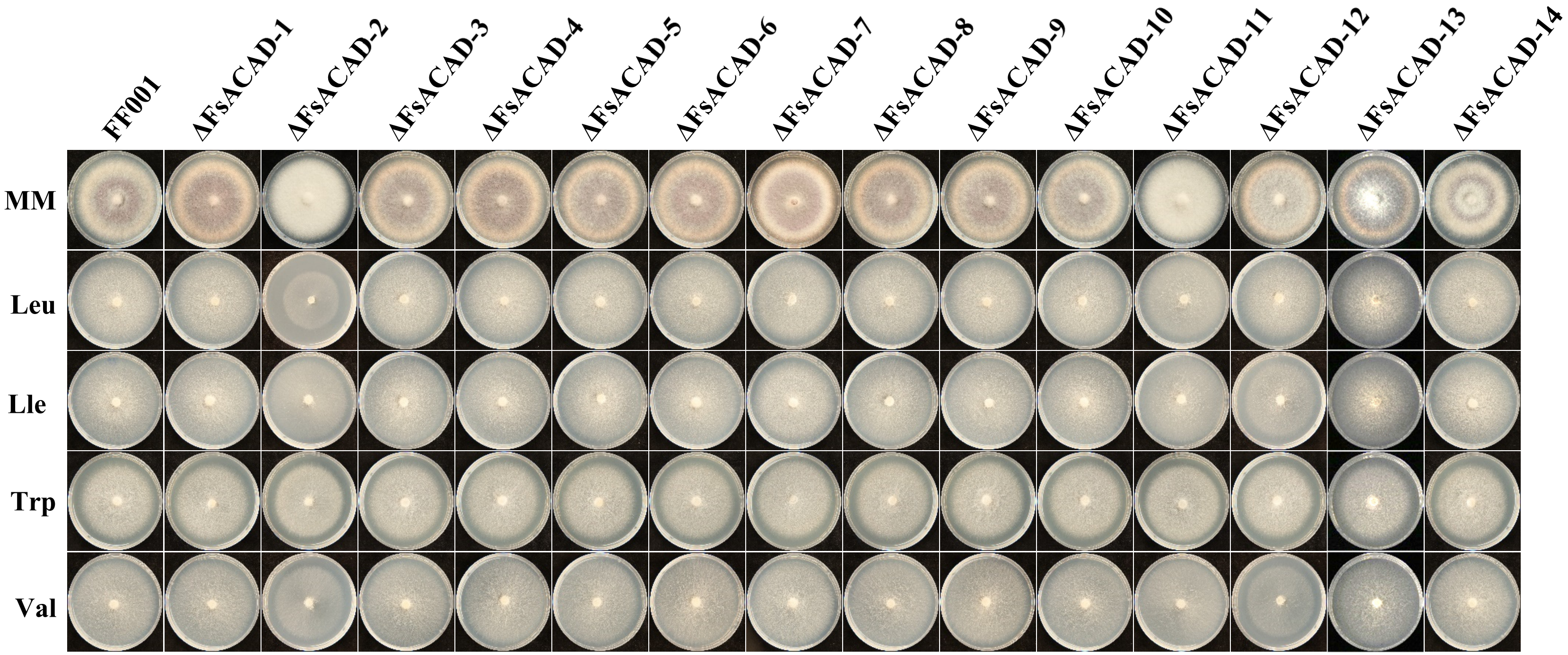

Figure S4. Morphology of *Fusarium sacchari* acyl-CoA dehydrogenase mutants (Δ*FsACADs*) in mediums with amine acid as sole carbon source. The petri dish is 90mm in diameter.

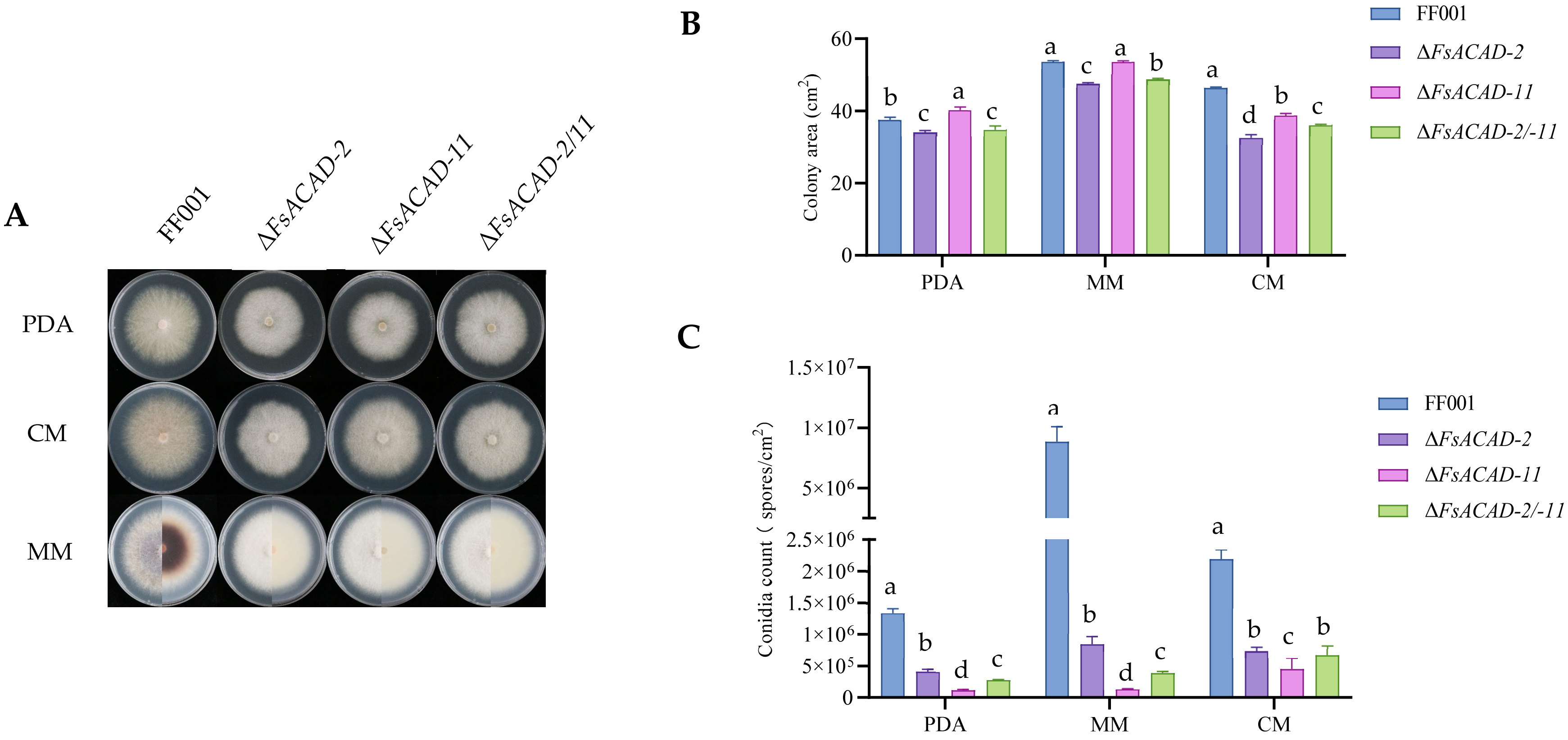

Figure S5. Phenotypes of *Fusarium sacchari* acyl-CoA dehydrogenases double knockout mutants in different mediums. (A) the front view of the colony on the PDA and CM plates, with the image depicts the front view of the colony, illustrating the morphology of aerial mycelium, juxtaposed with the rear view exhibiting the development of colony pigments on MM plates. The petri dish is 90mm in diameter. (B) the colony area of the *FsACAD* double knockout mutants. (C) the unit area conidia yield of the *FsACAD* double knockout mutants. Same letters indicate not significantly different ($p < 0.05$).

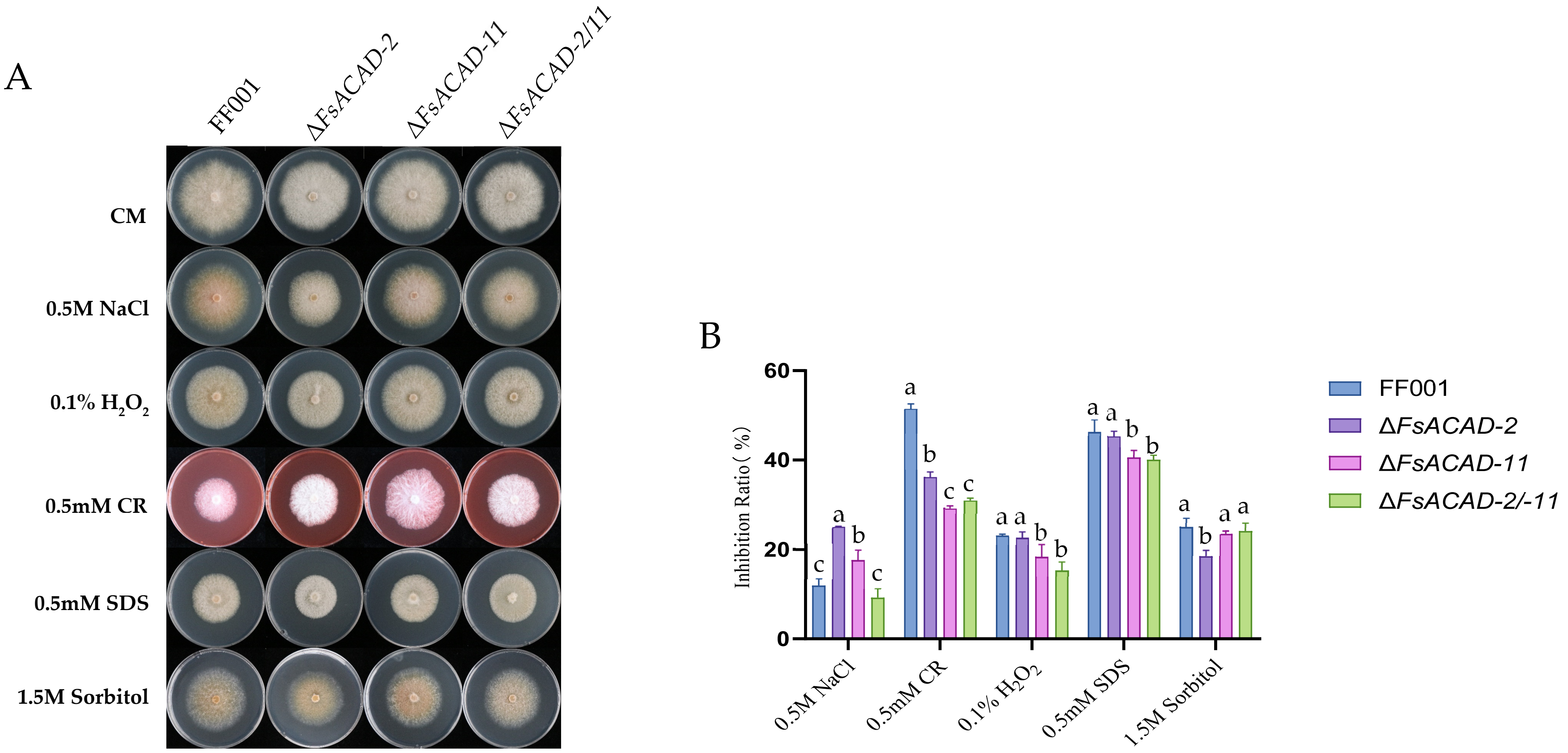

Figure S6. Stress response of *Fusarium sacchari* acyl-CoA dehydrogenases double mutant Δ*FsACAD-2/-11*. (A) Colony morphology on plates with stressors. The petri dish is 90mm in diameter. (B) Relative nhibition rates. Same letters indicate not significantly different ($p < 0.05$).

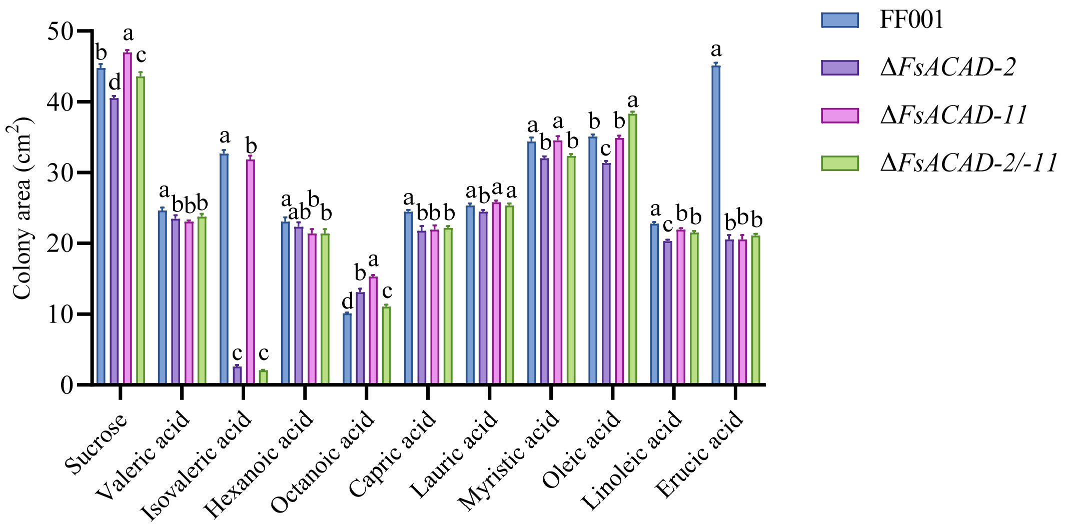

Figure S7. Colony size of *Fusarium sacchari* acyl-CoA dehydrogenases double mutant Δ*FsACAD-2*/*-11* on different mediums with various of fatty acids. Same letters indicate not significantly different ($p < 0.05$).

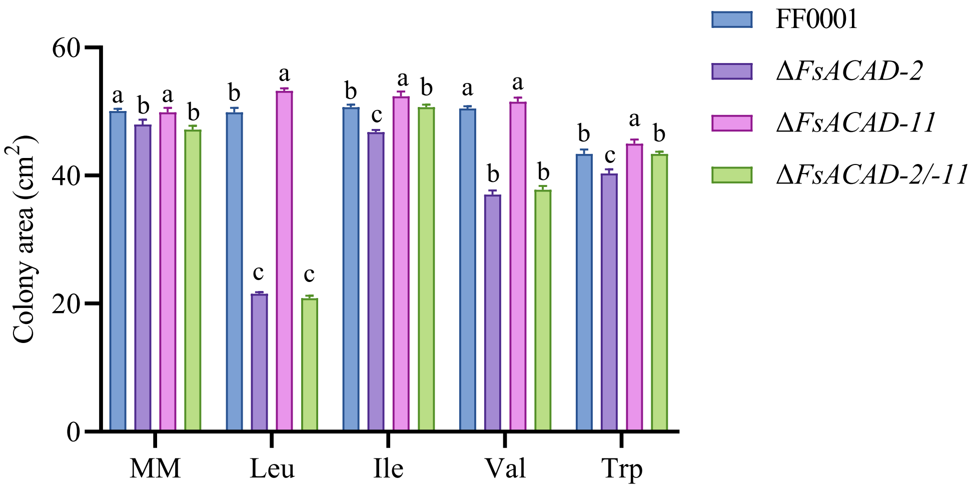

Figure S8. Colony size of the *Fusarium sacchari* acyl-CoA dehydrogenases double mutant Δ*FsACAD-2/-11* mutants in mediums with amine acid as sole carbon source. Same letters indicate not significantly different ($p < 0.05$).

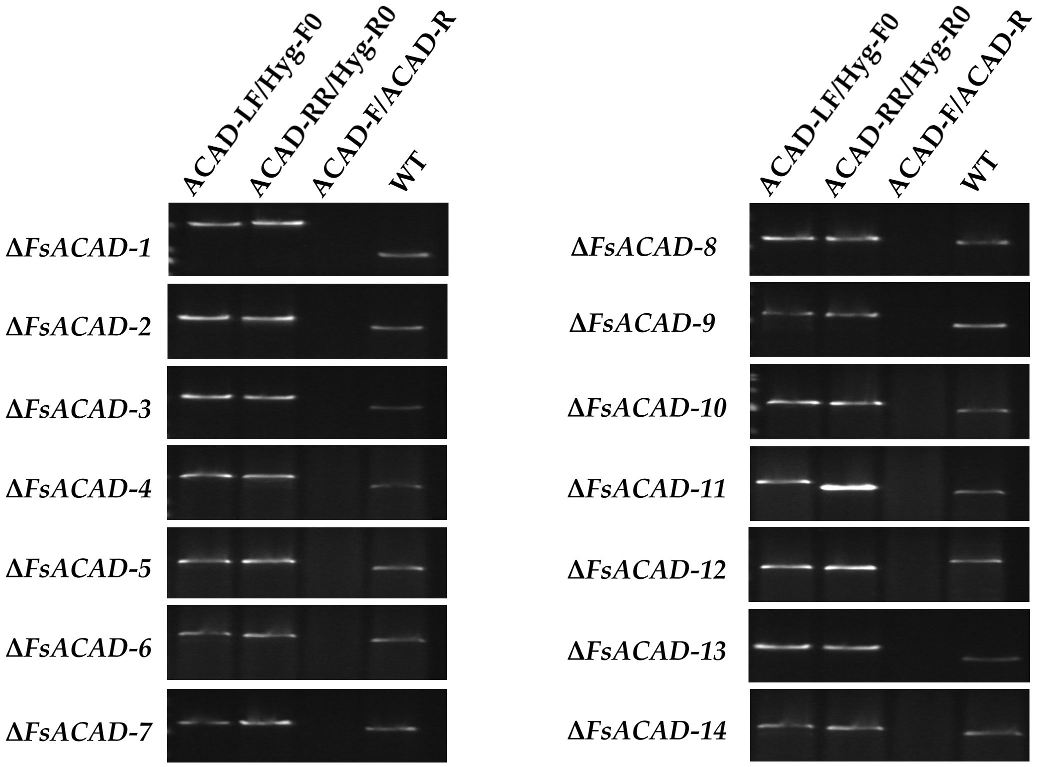

Figure S9. Validation of *Fusarium sacchari* acyl-CoA dehydrogenase mutants by PCR analysis. The upstream and downstream fragments containing part of the HYG gene were amplified using primers ACAD-LF/Hyg-F0 and ACAD-RR/Hyg-R0, respectively. The target gene was amplified using primers ACAD-F/ACAD-R, with the wild-type (WT) as the control (the primers ACAD-LF/RR and ACAD-F/R for each gene were different and specific).
